# A plant-derived antimicrobial peptide with multiple mechanisms of action exhibiting antibacterial and antibiofilm activities comparable to or superior to polymyxin B

**DOI:** 10.1016/j.crmicr.2025.100535

**Published:** 2025-12-17

**Authors:** Mohamad Anas Al Bouni, Rui M. Lima, Sándor Jenei, Hilda Tiricz, Edit Tímár, Ildikó Domonkos, Éva Kondorosi, Gabriella Endre

**Affiliations:** a Institute of Plant Biology, HUN-REN Biological Research Centre, Szeged, Hungary; b Doctoral School of Biology, Faculty of Science and Informatics, University of Szeged, Szeged, Hungary

**Keywords:** Antimicrobial peptide (AMP) NCR169C_17–38_, Biofilm eradication, Surpassed polymyxin B, Membrane permeabilization, Lipid binding, Nucleic acid interactions, Transcriptomic analysis

## Abstract

Antimicrobial peptides (AMPs) represent promising alternatives to conventional antibiotics in the fight against multidrug-resistant pathogens. Here, we characterize the antibacterial properties and molecular mode of action of the C-terminal fragment of the *Medicago truncatula* nodule-specific cysteine-rich peptide NCR169 (NCR169C_17–38_). This peptide exhibits strong bactericidal activity against a broad panel of Gram-positive and Gram-negative pathogens, including members of the ESKAPE group (*Enterococcus faecalis, Staphylococcus aureus, Klebsiella pneumoniae, Acinetobacter baumannii, Pseudomonas aeruginosa, Escherichia coli)*. NCR169C_17–38_ acted rapidly by permeabilizing bacterial membranes and causing severe morphological damage, outperforming polymyxin B in the rate of membrane disruption. The peptide bound selectively to anionic bacterial lipids such as cardiolipin and displayed remarkable thermal and storage stability. Moreover, NCR169C_17–38_ effectively inhibited biofilm formation and eradicated pre-formed biofilms of *Acinetobacter baumannii* even at sub-MBC concentration (3.2 μM), a feature not observed for polymyxin B. Transcriptomic analysis of *E. coli* exposed to sublethal peptide doses revealed a global shutdown of bacterial metabolic and genetic functions, with 450 of 503 differentially expressed genes being downregulated. Strong repression of genes involved in translation, energy production, and cell envelope biosynthesis was accompanied by upregulation of stress- and rescue-related genes linked to membrane repair, ion transport, and oxidative stress responses. Together, these results demonstrate that NCR169C_17–38_ exerts multifaceted antimicrobial effects - disrupting membranes, binding nucleic acids, and inducing transcriptional collapse - while maintaining its stability and biocompatibility. Together with its previously established antifungal activity and lack of mammalian cytotoxicity, NCR169C_17–38_ represents a compelling candidate for development as a next-generation antimicrobial agent.

## Highlights

• Potent antibacterial activity against eight pathogens across different growth media.
• Strong biofilm inhibition and bactericidal efficacy on biofilm-embedded cells.
• Biofilm eradication at sub-MBC outperforming polymyxin B.
• Lipid and nucleic acid binding, suggesting multiple intracellular modes of action.
• Global transcriptomic changes including membrane, stress, and metabolism genes.

## Graphical abstract

**Figure:**
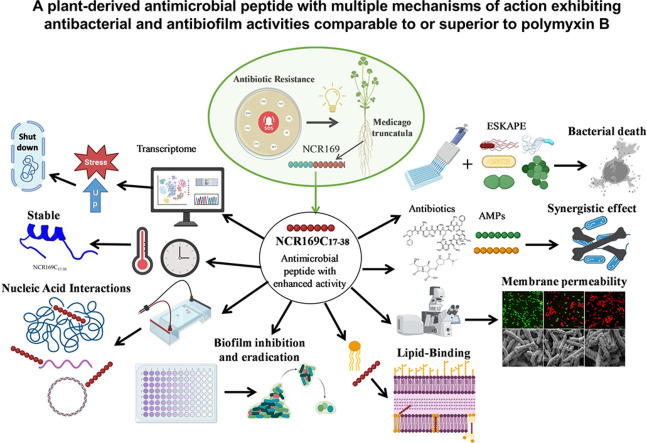

## 1 Introduction

Antimicrobial peptides (AMPs) represent effectors of the innate immune system in different organisms. AMPs exhibit a wide range of antimicrobial activity against bacteria, fungi, parasites and viruses, thus making them good candidates for new antimicrobial agents. The discovery of antibiotics revolutionized medicine, saving countless lives and contributing to the rapid expansion of the human population. However, their excessive and improper use has led to the emergence and widespread proliferation of antibiotic-resistant bacteria. Antibiotic resistance easily propagates via horizontal gene transfer making the antibiotics ineffective, which causes the death of hundreds of thousands of people each year by drug resistant microbial infections. Therefore, there is a great need for novel types of antimicrobial agents that are effective against the antibiotic resistant pathogens and can kill the most resilient ESKAPE bacterial strains; *Enterococcus faecalis, Staphylococcus aureus, Klebsiella pneumoniae, Acinetobacter baumannii, Pseudomonas aeruginosa* and *Enterobacter* species, which are inherently resistant to multiple antibiotics.

AMPs are produced by all living organisms and show extraordinary variety, with their variable sequence length - usually from 10 up to 60 amino acid residues - and high diversity in their amino acid composition and sequence, resulting in often broad-spectrum antimicrobial activity. AMPs are predominantly cationic peptides, but beside the positive charge their hydrophobicity and amphipathic nature also contribute to their antimicrobial properties (Bahar and Ren, 2013; Mirski et al., 2018). The structure of AMPs can be α-helical, β-sheet, cyclic or extended. The latter ones have no structural motifs but can contain specific amino acids in higher amounts. The most prevalent killing mechanisms of AMPs rely on their direct interaction with the bacterial membranes leading to the loss of membrane potential, altered membrane permeability, and metabolic leakage (Spindler et al., 2011; Huang and Li, 2023). However, certain AMPs enter the bacterial cell without membrane damage. The interaction of AMPs with intracellular targets is another mode of their killing mechanisms. The advantage of AMPs is that they have different bacterial targets as the classical antibiotics and have multiple mechanisms for killing. The multiple bacterial targets and their effects on distinct pathways make less likely the propensity for development of resistance that would require multiple mutations simultaneously (Brogden, 2005; Gan et al., 2021; Abbasi, 2025). Nevertheless, the proteolytic instability and toxicity of certain AMPs in human cells can be their shortcomings in applications (Marr et al., 2006; Yang et al., 2025).

The Antimicrobial Peptide Database (https://aps.unmc.edu) currently lists over 5000 AMPs, yet only 268 of them are of plant origin, despite plants representing an exceptionally rich and diverse source of such molecules. In plants, AMPs are mainly known as defensive peptides of the plant's innate immunity, functioning against a variety of pathogens. (Campos et al., 2018; Das et al., 2019). Besides them, a special group of AMPs has been identified in *Medicago truncatula* and related legumes (Lima et al., 2022), among the Nodule-specific Cysteine-Rich (NCR) peptides. These NCR peptides act as key regulators of symbiosis with rhizobial partners, controlling the differentiation of intracellular bacteria within root nodules. In *M. truncatula*, >700 genes encode NCR peptides, which are exclusively expressed in symbiotic nodule cells (Mergaert et al., 2003). NCRs drive the terminal differentiation of rhizobia into enlarged, polyploid, non-cultivable nitrogen-fixing bacteroids characterized by extensive changes in morphology, physiology, and membrane permeability (Van de Velde et al., 2010; Farkas et al., 2014). Mature NCR peptides are typically 30–50 amino acids long and contain four or six conserved cysteines embedded in highly variable sequences, resulting in a broad range of physicochemical properties and isoelectric points (pI 3.2–11.2) (Lima et al., 2020). Because NCR peptides irreversibly block bacterial cell division, many have been examined for antimicrobial activity, and several have demonstrated bactericidal effects against diverse pathogens (Tiricz et al., 2013; Balogh et al., 2014; Ordögh et al., 2014; Maróti et al., 2015; Farkas et al., 2017; Lima et al., 2022). Some of these cationic NCRs were shown to permeabilize bacterial membranes, induce propidium iodide uptake, and depolarize membrane potential, leading to cell death (Mikuláss et al., 2016).

Among them, so far NCR247 was the best-characterized example. This 24-amino-acid cationic peptide (pI 10.15) contains four cysteines and exhibits remarkable protein-binding capacity, affecting transcription, translation, and cell division in *Sinorhizobium meliloti* through direct interaction with FtsZ (Tiricz et al., 2013; Farkas et al., 2014; Penterman et al., 2014). Shorter NCR247 derivatives and chimeric peptides have proven even more potent, killing ESKAPE pathogens at submicromolar concentrations (1.6 – 3.2 µM) without cytotoxic effects on human cells (Jenei et al., 2020).

NCR169 is another NCR peptide essential for differentiation of nitrogen fixing bacteroids in *Medicago* species (Horváth et al., 2015). This 38-amino-acid peptide contains four cysteines that are critical for its symbiotic function *in planta*. Screening of NCR169 and 18 other NCR peptides with diverse physicochemical features against *Candida albicans* revealed that only those with a pI above 9.5 inhibited fungal growth, accordingly NCR169 (pI 8.6) was inactive in this assay (Ördögh et al., 2014). On the other hand, its C-terminal derivative NCR169C_17–38_ (pI 10.48) demonstrated potent antifungal activity against several *Candida* species (Szerencsés et al., 2021) and *Cryptococcus neoformans* (Szerencsés et al., 2025), and against ESKAPE bacteria as well (Howan et al., 2023).

In the present study, we systematically evaluated the antimicrobial activity of NCR169, its cysteine variants, and a series of shorter fragments - including the potent derivative NCR169C_17–38_ - against a panel of clinically relevant bacterial pathogens, including ESKAPE species. Through a combination of structural, biochemical, and transcriptomic approaches, we defined the region critical for the reliable antimicrobial activity, elucidated its dual mechanism of action involving membrane disruption and transcriptional suppression, and characterized the bacterial stress responses elicited upon exposure. Together, these findings provide mechanistic insight into NCR169C_17–38_ function and establish this peptide as a potent and versatile candidate for next-generation antimicrobial development.

## 2 Materials and methods

### 2.1 Peptide synthesis, bacterial strains, and antimicrobial assays

NCR169, NCR169C_17–38_ and derivatives were synthesized by standard Fmoc solid-phase peptide synthesis (SPPS) on a TentaGel S RAM resin using an automated peptide synthesizer (CEM Liberty Blue) followed by RP-HPLC purification and ESI-MS confirmation of identity (Jenei et al., 2020; Howan et al., 2023).

The Gram-positive strains *Enterococcus faecalis* (ATCC 29212), *Staphylococcus aureus* (HNCMO112011), and *Listeria monocytogenes* (ATCC 19111), and the Gram-negative strains *Pseudomonas aeruginosa* (ATCC 27853), *Escherichia coli* (ATCC 8739), *Salmonella enterica* (ATCC 13076), *Klebsiella pneumoniae* (NCTC 13440), and *Acinetobacter baumannii* (ATCC 17978) were obtained from ATCC (United States) and NCTC (National Collection of Type Cultures – England).

Minimal bactericidal concentrations (MBC) of peptides and reference antibiotics were determined in 20 mM potassium phosphate buffer (PPB, pH 7.4) and Mueller–Hinton broth (MHB) as described by (Jenei et al., 2020). Briefly, bacterial cultures were grown to mid-log phase, washed, adjusted to OD₆₀₀ = 0.1 (∼10⁷ CFU/mL), added 10 μl of it into 100 μl mixture and incubated in 96-well plates with 2-fold serial dilutions of peptides or antibiotics. After incubation at 37 °C with shaking, bacterial viability was assessed by plating 5 μL aliquots on LB agar. MBC was defined as the lowest concentration yielding no colony growth. Experiments were done at least in three biological replicates.

### 2.2 Combined treatment of *E. coli* with NCR169C_17–38_ and antibiotics or NCR335 fragments

The effects of the different combinations of NCR169C_17–38_ with streptomycin, meropenem, polymyxin B and fragments of NCR335 (NCR335N_1–19_: RLNTTFRPLNFKMLRFWGQ; NCR335C_13–33_: KDCPKLRRANVRCRKSYCVPI) were determined by standard checkerboard titration method. Streptomycin was tested at concentrations ranging from 25 μM to 0.4 μM, meropenem from 200 μM to 3.1 μM, and polymyxin B from 6.3 μM to 0.1 μM. NCR335N_1–19_ was tested in a concentration range from 3.1 μM to 0.05 μM, while NCR335C_13–33_ from 12.5 μM to 0.2 μM. NCR169C_17–38_ concentrations ranged from 25 μM to 0.4 μM when tested with antibiotics, whereas in combinations with peptide fragments, lower concentration of NCR169C_17–38_ (3.1 μM to 0.05 μM) were applied. The initial *E. coli* density in each well were adjusted to OD_600_ = 0.01 corresponding to ∼10^6^ bacteria/ml. A volume of 45 μl of antibiotic dissolved in MHB medium was combined with 45μl of NCR169C_17–38_ or NCR335 fragments dissolved in PPB were mixed with 10 μl of bacterial suspension and incubated for 3 h at 37 °C with 250 rpm shaking, 5 μl from each sample was placed on LB agar and MBC was detected after overnight incubation at 37 °C. The effectiveness of combination treatments was measured using the fractional bactericidal concentration (FBC) index, which is a metric used to assess the interaction between antimicrobial agents, determining whether their combined effect is synergistic, additive, or antagonistic. First, the MBC values of polymyxin B, meropenem, streptomycin, NCR335N_1–19_, and NCR335C_13–33_ were determined in *E. coli*. Subsequently, their combinations with NCR169C_17–38_ were examined using the FBC index. FBC = FBCA + FBCB. FBCA = (MBCA in combination) / (MBCA alone); FBCB = (MBCB in combination) / (MBCB alone). Experiments were done in three biological replicates.

### 2.3 Biofilm inhibition and biofilm eradication assays on *Acinetobacter baumannii*

For the biofilm inhibition assay the antimicrobial activity test was done in 1/10 MHB using *A. baumannii* treated with the respective dilutions of NCR169C_17–38_, meropenem or polymixin B for 24 h. After taking the 5 μl samples at the end of the treatment and placed on LB agar to detect bacterial growth, the rest of the grown bacterial suspensions were discarded from the plate and the remaining biofilm on the walls of the plate was then washed three times with sterile water and stained using 1 % Crystal Violet (CV) for 15 min to visualize the produced biofilm. The non-adherent CV dye was then removed by washing with water three times. The plate was left for complete drying, then the biofilm-bound CV dye was dissolved in 200 µl of 30 % acetic-acid with shaking over 15 min. The absorbance was measured at OD_550_ using a HIDEX Plate Reader (Hidex Sense Microplate Reader with Plate Reader Software version 5064, Hidex, Finland). All measurements were repeated three times.

For the biofilm eradication assay the difference was at the beginning of the experiments when bacteria were pre-cultured for 24 h or 72 h in MHB with a starting OD_600_ = 0.01. Then bacterial suspensions were removed from the wells followed by washing three times with 200 µl of 1/2 MHB. Subsequently fresh 1/10 MHB was added to the wells containing the respective dilutions of NCR169C_17–38_, meropenem or polymixin B and the treatment lasted either 3 h or 24 h. Experiments were done in three biological replicates.

### 2.4 Cell membrane damage assessed by different methods

Microbial cell membrane integrity or damage was assayed with Live/Dead staining. *E. coli* were grown as for the antimicrobial activity assay and washed with PPB. Cells were resuspended in PPB, and diluted to OD_600_ = 0.1 and then treated with 3.125 or 1.6 μM of NCR169C_17–38_ at room temperature. Untreated cells served as negative control. Cells were subsequently stained with 5 μM SYTO 9 and 5 μM propidium iodide (PI). After a 10-minute incubation in the dark, 5 µl of each cell suspension was placed on a microscope slide, covered with thin 2 % (w/v) agar slices, and examined using an Olympus Fluoview FV1000 confocal laser microscope at × 60 magnification. For SYTO 9, excitation was at 488 nm and emission was collected between 500 and 530 nm; for PI, excitation was at 543 nm with emission detected between 555 and 655 nm. Sequential scanning was used to avoid crosstalk of the fluorescent dyes.

To visualize morphological changes on the bacterial surface Log phase OD_600_ = 0.1 *E. coli* bacteria were treated with NCR169C_17–38_ peptide at concentrations of 1.6 µM and 3.1 µM in PPB. After treatment, the samples were fixed with 2.5 % (v/v) glutaraldehyde in phosphate buffered saline (PBS, pH 7.4). 5 μl of the above bacterial suspensions were spotted on a 0.1 µm PC membrane (Merck Isopore). The membranes were washed twice with PBS and gradually dehydrated with ethanol series (30, 50, 70, 80 %, and three times 100 % ethanol, each for minimum 30 min). The samples were dried with a critical point dryer (K850: Quorum Technologies Ltd.), followed by 12 nm gold coating and observed under a JEOL JSM-7100F/LV scanning electron microscope.

To monitor PI uptake in real time, 10 µl of *E. coli* suspension (OD₆₀₀ = 0.1, prepared as described above) was mixed with 90 µl of PPB containing a two-fold serial dilution of NCR169C_17–38_ (12.5 µM to 0.8 µM) or polymyxin B (0.8 µM), along with 200 ng/ml PI. Fluorescence was recorded using a Hidex Sense Microplate Reader (software version 5064) with excitation and emission wavelengths set at 535 nm and 617 nm, respectively. Experiments were repeated three times.

### 2.5 The peptide heat stability and serum stability assays

For heat stability test serial dilutions of the NCR169C_17–38_-StrepII peptide were prepared in PPB at concentrations of 25, 12.5, 6.3, 3.1 µM, following the same protocol used in the antimicrobial assay. One set of samples was incubated overnight at 37 °C with shaking, another set was subjected to heat stress at 80 °C for 20 min, while one set was freshly prepared. Dot-blot analysis was performed by spotting 5 µl of each peptide solution onto a 0.22 µm nitrocellulose membrane (Bio-Rad). Detection of the StrepII-tagged peptide was carried out using the Precision Protein StrepTactin-HRP conjugate (Bio-Rad), following the manufacturer’s recommended protocol. Signal development was achieved using the Clarity Western ECL Substrate (Bio-Rad), with the membrane incubated for 5 min at room temperature, followed by exposure to Fuji Medical X-ray film for visualization. The same sets of serial dilutions were also used in antimicrobial test against *E. coli* to determine their MBCs.

For serum stability test human blood was purchased from the Regional Blood Centre in Szeged (the use of human blood has been authorized by the Regional Hungarian Ethics Committee and approved by the Ethics Review Sector of DG RTD (European Commission) in connection with EK’s ERC AdG SymBiotics). The cells from 10 ml of EDTA-blood were centrifuged at 1500 × *g* for 1 min and washed several times in TBS buffer (10 mM Tris, pH: 7.2, 150 mM NaCl) until the supernatant, representing the serum, became colorless. The NCR169C_17–38_ peptide was added to the serum at 1:1 ratio and incubated at room temperature for 8 h. Subsequently, serial 2-fold dilutions of the peptide were prepared in PPB, with concentrations ranging from 100 to 0.2 µM and antimicrobial assay was done to determine the MBC values. Experiments were done in three biological replicates.

### 2.6 Lipid overlay assays

Membrane lipid strips (Echelon Biosciences, P-6002) were incubated for 1 h in blocking solution (3 % BSA in TBS-Tween 0.1 %) with gentle agitation followed by four 8-min washes in TBS-Tween. Then, strips were incubated with 2 μM of NCR169C_17–38_ in blocking solution for 1 h. Not bound peptide was washed off by four 8-min washes in TBS-Tween. The bound StrepII-tagged peptide was then detected on the strips using the same procedure described for the dot blot, except that chemiluminescence was visualized with an iBright™ FL1500 Imaging System (Invitrogen).

### 2.7 Liposome sedimentation assays

To prepare the liposomes, phospholipids DMPC and Cardiolipin purchased from Avanti Polar Lipids (Alabaster, AL, USA) were dissolved in Chloroform:Methanol:Water in the ratio of (65:25:4) and (73:23:3) respectively, and dried under vacuum for 1 h. The lipid film was suspended in the assay buffer (20 mM HEPES, 150 mM NaCl, pH 7.0), vortexed, and sonicated for 15 min to form the liposomes. The assay was carried out by incubating 2 mM of the liposomes with 20 μM of NCR169C_17–38_ peptide and incubated for 30 min at room temperature, followed by ultracentrifugation at 200,000 × *g* for 15 min at 20 °C. After centrifugation, the supernatant (sn) and pellet (p) fractions were collected separately and were subjected to glycine-SDS-PAGE. The peptides were detected by Coomassie Brilliant Blue staining.

### 2.8 Microscale thermophoresis assay

Binding interactions were analyzed using a Monolith NT.115 microscale thermophoresis (MST) instrument (NanoTemper Technologies, Munich, Germany). The instrument was equipped with a capillary tray, enabling successive measurements within each run using coated capillaries. For the binding check assays, measurements were performed with four samples, while for the affinity determination assays, sixteen samples were analyzed. Data acquisition and processing were conducted with the NT Analysis software (NanoTemper Technologies). All experiments were carried out under optimized MST settings, with the LED excitation power set to 100 % and the MST laser power maintained at the medium-power setting. NCR169C_17–38_–5-FAM (5-Carboxyfluorescein) has a green fluorescence and diluted in Tris buffer supplemented with 100 mM NaCl and 0.05 % Tween-20. Lipids films were dissolved in the same buffer. Cholesterol (Thermo Scientific) was first dissolved in chloroform, dried for 1 hour, and then incorporated into DMPC liposomes at a 1:1 ratio by rehydration in the liposome solution.

### 2.9 Nucleic acid binding assays

The DNA- and RNA-binding abilities of NCR169C_17–38_, its parental peptide NCR169, and two shorter derivatives (NCR169_16–27_ and NCR169_17–27_) were initially evaluated using a gel retardation assay. In this setup, 100 ng of either *E. coli* genomic DNA or total RNA was incubated with 10 µM or 100 µM of each peptide for 30 min at 37 °C. Reaction mixtures, along with untreated controls, were then subjected to agarose gel electrophoresis, and the resulting nucleic acid migration patterns were examined for changes indicative of peptide binding. The interaction of NCR169C_17–38_ with circular plasmid DNA (pDEST-GBKT7) was also characterized using peptide concentrations ranging from 50 µM to 0.8 µM, prepared by 2-fold serial dilution. Experiments were done in three biological replicates.

In addition, DNA-binding kinetics were monitored in real time using a Hidex Sense plate reader at room temperature in three independent experiments. Reaction mixtures contained 100 ng of *Hin*dIII-digested lambda phage DNA in 1 × SYBR Gold (Thermo Scientific), either without peptide or with 10 µM of NCR169C_17–38_ (2.74041 µg), NCR169 (4.56545 µg), NCR169_16–27_ (1.56597 µg), or NCR169_17–27_ (1.40279 µg). Peptides were added either at the start (0 min) or at 14 min. Fluorescence from DNA-bound SYBR Gold was measured every 3 min (excitation 490 nm, emission 535 nm). At 27 min, proteinase K (1 mg/ml; 100 µg final) was added to peptide-treated samples to assess the effect on peptide–DNA binding. Experiments were done in three biological replicates.

For localization experiment treatment of the *E.coli* cells with the 5-carboxyfluorescein–labeled peptide (NCR169C_17–38_–5-FAM) was done as described before for the microbial cell membrane integrity assay. The Hoechst 33342 staining was used at 10 µg/mL concentration and cells were checked with The Stellaris 8 confocal microscope at × 63 magnification. For Hoechst 33342, excitation was at 361 nm and emission was collected between 430–500 nm; for 5-FAM-labeled peptide, excitation was at 488 nm with emission detected between 555–655 nm.

### 2.10 Preparation of RNA samples for transcriptome analysis

*E. coli* starter cultures in the exponential growth phase were diluted to OD_600_ = 0.05 and grown to early logarithmic phase (OD_600_ = 0.2) in six separate flasks every 10–10 ml in LSM (MBC = 6.2 µM) liquid medium (Farkas et al., 2018). Three biological replicates were treated at OD_600_ = 0.2 with a final concentration of 1.6 µM NCR169C_17–38_ peptide for 20 min, while the control samples were treated with the same amount of water for 20 min. Total bacterial RNA was purified from samples using the RNeasy Mini Kit/QIAGEN (cat. no. 74104) following the instructions in the protocol, prior the purification with treatment of RNAprotect Bacterial Reagent/QIAGEN (cat. no. 76506). Residual DNA was eliminated using Ambion DNase I (RNase-free). The samples were precipitated with ethanol and resuspend in RNase free water. Quality and quantity was detected using 2200 TapeStation (Agilent) and Qubit RNA BR Assay (Sigma)

### 2.11 Transcriptome sequencing by RNA-Seq methods

Sequencing libraries were prepared for the transcriptome samples using Zymo-Seq RiboFree Total RNA Library Kit. Sequencing was carried out by Seqomics company. Paired-end fragment reads were generated on an Illumina NextSeq sequencer using NextSeq® 500/550 High Output Kit v2 (300 cycles). Primary data analysis (base-calling) was carried out with “bcl2fastq” software (v2.17.1.14, Illumina).

### 2.12 Validation of the mRNA sequencing data

To validate the sequencing results, quantitative reverse transcription PCR (qRT-PCR) was performed on selected genes (*cysG, hcaT, osmB, rplK, ompF, rpsJ, tufAB, ihfB, rpoA, metK, lpp*) by using the primers shown in Supplementary Table 2. Data were normalized to *cysG* gene that proved to be a reliable reference gene during stress conditions (Zhou et al., 2011; Peng et al., 2014), and the results were compared to the sequencing data. RNA was reverse transcribed by the high-capacity cDNA reverse transcription kit (Applied Biosystems). PCR amplification was performed using the Power Up SYBR green kit (Applied Biosystems) and detected by the incorporation of the SYBR green dye in a StepOne Plus (Thermo Fisher) real-time PCR device using StepOne software version 2.1 (Life Technologies). Two technical replicates were performed on all biological replicates.

### 2.13 Bioinformatics analysis

PM4NGS framework (Vera Alvarez et al., 2021) was used for processing and analyzing the samples. Samples quality control was checked using the tool FastQC (Andrews, 2018). After this step, a required trimming was executed reducing distortions on the “per base sequence content” at the begging of the reads using Trimmomatic (Bolger et al., 2014). The trimmed samples were aligned to the *E. coli* ATCC_8739 genome database using STAR (Dobin et al., 2013) as described by (Baruzzo et al., 2017; supplementary Table 37). Reads were required to be uniquely mapped using Samtools (Li et al., 2009) for filtering, sorting and indexing. RSeQC (Wang et al., 2012) was used to assess quality of each sample. The RNA sequencing abundance, raw read counts and TPM values, was calculated with TPMCalculator (Vera Alvarez et al., 2019) using the default parameters. The differential gene expression analysis was executed using the Bioconductor packages Deseq2 (Love et al., 2014) and EdgeR (Robinson et al., 2010). An adjusted p-value of 0.05 and a logarithmic fold change of 2.0 was used to identify differentially expressed genes. The KEGG pathways analysis was performed with the web tool ShineyGO 0.85 (Ge et al., 2020; Kanehisa et al., 2023).

The GO enrichment analysis was executed using the Python package goenrichment, version 1.0.3, (https://pypi.org/project/goenrichment/). This package uses the hypergeometric distribution for calculating p-values and the Benjamini/Hochberg for p-value correction. The final list of the differentially expressed genes was selected from the interception of both programs. All tools were executed using publicly available Common Workflows Language (CWL) files and docker images with fixed tool versions.

## 3 Results

### 3.1 pH-Dependent solubility and bactericidal activity of NCR169 and its structural variants

NCR169, its oxidized form (NCR169ox), and its variant in which the four cysteines were replaced with serines (NCR169-C_9,15,28,33_/S) (Table 1.A) were chemically synthesized with C-terminal amidation. These peptides were tested for antimicrobial activity against a panel of ESKAPE pathogens - *Enterococcus faecalis, Staphylococcus aureus, Klebsiella pneumoniae, Acinetobacter baumannii, Pseudomonas aeruginosa, E. coli* - as well as *Listeria monocytogenes* and *Salmonella enterica.* Peptides were applied to 10^6^ log-phase bacterial cultures in a two-fold dilution series (25 - 0.1 µM) in potassium phosphate buffer (PPB) at pH 7.4. After three hours of incubation, 5 µl of each treatment was plated on LB agar and incubated overnight at 37 °C to assess bacterial survival. Minimal bactericidal concentrations (MBCs), defined as the lowest concentration eliminating bacterial growth, are presented in Table 1B.

**Table 1:** Minimal bactericidal concentrations (MBC) of NCR169 and its derivatives against *Enterococcus faecalis (E.f.); Staphylococcus aureus (S.a.); Klebsiella pneumoniae (K.p.); Acinetobacter baumannii (A.b.); Pseudomonas aeruginosa (P.a.); Escherichia coli (E.c.); Listeria monocytogenes (L.m.); Salmonella enterica (S.e.)*. A. Amino acid sequence and isoelectric points (pI values) of the peptides. B. MBC values of the peptides (in μM) in PPB at pH 7.4 and 5.8 (in bold letters) after three hours of treatment.

**A**									
Peptides	Amino Acid Sequence							pI	
NCR169	EDIGHIKYCGIVDDCYKSKKPLFKIWKCVENVCVLWYK							8.6	
NCR169ox	EDIGHIKYCGIVDDCYKSKKPLFKIWKCVENVCVLWYK							8.6	
NCR169-C_9,15,28,33_/S	EDIGHIKY**S**GIVDD**S**YKSKKPLFKIWK**S**VENV**S**VLWYK							9.7	
**B**									
Peptides	pH	*E. f.*	*S. a.*	*K. p.*	*A. b.*	*P. a.*	*E. c.*	L. *m.*	*S. e.*
NCR169	7.4	-	6.3*	12.5*	3.1	12.5*	3.1*	12.5*	3.1*
NCR169ox	7.4	-	25	25	1.6	6.3	6.3	12.5	12.5
NCR169-C_9,15,28,33_/S	7.4	-	-	-	3.1	25	6.3	-	-
**NCR169**	**5.8**	**-**	**3.1**	**12.5**	**3.1**	**6.3**	**3.1**	**6.3**	**3.1**
**NCR169ox**	**5.8**	**-**	**3.1**	**3.1**	**3.1**	**6.3**	**1.6**	**3.1**	**3.1**
**NCR169-C_9,15,28,33_/S**	**5.8**	**-**	**6.3**	**12.5**	**3.1**	**6.3**	**1.6**	**25**	**6.3**

-: lack of bactericidal activity up to 25 µM. * The best MBC value measured but there were more than two fold dilution differences in the replicates.

NCR169 demonstrated potent bactericidal activity at 3.1 µM against *A. baumannii, E. coli* and *S. enterica*; 6.3 µM against *S. aureus* and 12.5 µM against *K. pneumoniae, P. aeruginosa* and L. *monocytogenes,* while showing no detectible activity against *E. faecalis* (Table 1.B). These values represent the average of four biological replicates, although significant variability was observed, likely due to the poor solubility of NCR169 at pH 7.4, indicated by a visible white turbidity upon peptide addition. In contrast, both NCR169ox or NCR169-C_9,15,28,33_/S exhibited no visible turbidity and yielded consistent MBC values at this pH. While NCR169ox showed similar or slightly reduced antimicrobial activity compared to NCR169, NCR169-C_9,15,28,33_/S variant demonstrated markedly weaker efficacy, with bactericidal effects restricted to *A. baumannii, P. aeruginosa*, and *E. coli*.

Using PPB at pH 5.8 resolved the solubility problem of NCR169, eliminating visible turbidity and producing reproducible MBC values: 3.1 µM against *S. aureus, A. baumannii, E. coli* and *S. enterica;* and 6.3 µM against *P. aeruginosa* and L. *monocytogenes* (Table 1.B). Both the oxidized form of NCR169 and NCR169-C_9,15,28,33_/S were more active at pH 5.8 showing significantly lower MBC values against most bacteria than at pH 7.4. However, their activities were largely comparable to NCR169 under these acidic conditions. Notably, NCR169ox showed slightly enhanced activity against *K. pneumoniae,* whereas NCR169-C_9,15,28,33_/S was less effective against L. *monocytogenes*. These findings suggest that the cysteine residues in NCR169 are not essential for its antimicrobial activity at pH 5.8, although they may contribute to enhanced efficacy against specific strains such as L. *monocytogenes*.

### 3.2 NCR169C_17–38_: A C-Terminal segment with consistent and potent antibacterial activity

Next, various short derivatives of the NCR169 peptide (Table 2A) were tested for antimicrobial activity under different conditions. We have shown in previous works that the 22 amino acid long sequence at the C-terminal end, NCR169C_17–38_, was effective against several fungi, including *Candida* species (Szerencsés et al., 2021) and *Cryptococcus neoformans* (Szerencsés et al., 2025). Moreover, NCR169C_17–38_ and its substituted derivatives exhibited clear bactericidal effects against eight bacterial strains (Howan et al., 2023), also used in this study. Since adding an eight-residue StrepII-tag (WSHPQFEK) to other NCR peptides either preserved or enhanced their antimicrobial activity (Jenei et al., 2020), we tested a tagged version of NCR169C_17–38_ (NCR169C_17–38_-StrepII). A one-residue longer variant, NCR169_16–38_, which carries Tyr at position 16, was also included in the study. Additionally, four shorter fragments of NCR169C_17–38_ were tested, including NCR169_16–27_, previously shown to be active against *E. coli* and *S. meliloti* (Isozumi et al., 2021). As none of the peptides had solubility issues, the antimicrobial tests were performed in PPB pH 7.4.

**Table 2:** The antimicrobial activity of short derivatives of NCR169. A. Amino acid sequence and pI values of the short peptide fragments. B. MBC values of these peptides (in μM) in PPB (20 mM PPB at pH 7.4) or in HEPES buffer (5 mM HEPES pH 7.0). The MBCs of peptides are shown against *Enterococcus faecalis (Ef); Staphylococcus aureus (Sa); Klebsiella pneumoniae (Kp); Acinetobacter baumannii (Ab); Pseudomonas aeruginosa (Pa); Escherichia coli (Ec); Listeria monocytogenes (Lm); Salmonella enterica (Se)*.

**A**									
**Peptides**	**Amino Acid Sequence**							**pI**	
NCR169C_17–38_	KSKKPLFKIWKCVENVCVLWYK							10.2	
NCR169C_17–38_-StrepII	KSKKPLFKIWKCVENVCVLWYKWSHPQFEK							10.1	
NCR169_16–38_	YKSKKPLFKIWKCVENVCVLWYK							10.2	
NCR169_21–38_	PLFKIWKCVENVCVLWYK							9.1	
NCR169_27–38_	KCVENVCVLWYK							8.1	
NCR169_16–27_	YKSKKPLFKIWK							11.3	
NCR169_17–27_	KSKKPLFKIWK							14	
**B**									
**Peptides**	***Buffer***	***E. f.***	***S. a.***	***K. p.***	***A. b.***	***P. a.***	***E. c.***	**L. *m.***	***S. e.***
NCR169C_17–38_	PPB	6.3	3.1	3.1	3.1	3.1	1.6	3.1	3.1
NCR169C_17–38_-StrepII	PPB	3.1	3.1	3.1	3.1	3.1	1.6	3.1	3.1
NCR169_16–38_	PPB	3.1	3.1	3.1	3.1	3.1	1.6	3.1	3.1
NCR169_21–38_	PPB	-	-	-	12.5	-	25	-	-
NCR169_27–38_	PPB	-	-	-	-	-	25	-	-
NCR169_16–27_	PPB	-	-	-	-	-	25	-	-
NCR169_17–27_	PPB	-	-	-	-	-	-	-	-
NCR169C_17–38_	HEPES	3.1	3.1	6.3	1.6	3.1	1.6	3.1	3.1
NCR169_16–38_	HEPES	3.1	1.6	3.1	3.1	3.1	1.6	3.1	1.6
NCR169_16–27_	HEPES	-	3.1	6.3	3.1	3.1	3.1	12.5	3.1
NCR169_17–27_	HEPES	-	-	-	12.5	3.1	6.3	-	25

-: lack of bactericidal activity up to 25 µM.

NCR169C_17–38_ effectively killed the tested pathogens, as did both NCR169C_17–38_-StrepII and NCR169_16–38_ (Table 2B). Shorter fragments of NCR169C_17–38_, representing either its C-terminal (NCR169_21–38_ and NCR169_27–38_) or N-terminal (NCR169_16–27_ and NCR169_17–27_) segments were mostly ineffective at 25 µM, except against *E. coli*, for which MBCs reached up to 25 µM. Given that NCR169₁₆_-_₂₇ had previously shown strong activity against *E. coli* K12 strain in 5 mM HEPES buffer (pH 7.0) (Isozumi et al., 2021) we tested the activity of NCR169_16–27_, NCR169_17–27_, and the longer NCR169C_17–38_, NCR169_16–38_ peptides under the same conditions (Table 2B). MBC values for NCR169C_17–38_, NCR169_16–38_ remained similar to those in PPB. However, NCR169_16–27_ peptide - despite its poor performance in PPB - exhibited significant activity in HEPES, with MBCs comparable to those of NCR169C_17–38_, except for L. *monocytogenes* (12.5 µM) and *E. faecalis* (inactive). The absence of Tyr (NCR169_16_) in NCR169_17–27_, led to a loss of activity against four bacterial species and increased MBCs for *A. baumannii, E. coli* and *S. enterica*.

Further testing in Mueller Hinton Broth (MHB), which contains divalent cations known to reduce cationic AMP activity (Hancock and Sahl, 2006), revealed elevated MBCs (6.3 or 12.5 µM) for NCR169C_17–38_, NCR169C_17–38_-StrepII, and NCR169_16–38_. In this medium NCR169_17–27_ and NCR169_16–27_ were inactive at 25 µM (Table 3). However, the addition of 0.4 mM EDTA – without affecting bacterial viability - restored the activity of NCR169C_17–38_ and NCR169_16–38_ to PPB-level MBCs. EDTA had only a limited effect on NCR169_16–27_ and NCR169_17–27_, NCR169_16–27_ regained partial activity only against *E. coli* (MBC 6.3 µM). Based on the antimicrobial test results, the most reliable NCR169C_17–38_ was chosen for in-depth analysis and mode-of-action studies. Some key properties were compared with those of other peptide variants (Table 3).

**Table 3:** Minimal bactericidal concentrations (MBC; in µM) of the selected short peptides on three tested pathogens after 3 h of treatment in MHB in the absence (0) or presence (+) of 0.4 mM EDTA. *Escherichia coli (Ec), Acinetobacter baumannii (Ab), Staphylococcus aureus (Sa).*

Peptides	EDTA	*Ec*	*Ab*	*Sa*	EDTA	*Ec*	*Ab*	*Sa*
NCR169C_17–38_	**0**	6.3	6.3	12.5	**+**	1.6	3.1	3.1
NCR169C_17–38_-StrepII	**0**	12.5	6.3	25	**+**	1.6	3.1	3.1
NCR169_16–38_	**0**	6.3	6.3	25	**+**	1.6	1.6	3.1
NCR169_16–27_	**0**	-	-	-	**+**	6.3	-	25
NCR169_17–27_	**0**	-	-	-	**+**	-	-	-

-: lack of bactericidal activity up to 25 µM.

### 3.3 Synergistic effect of NCR169C_17–38_ peptide with conventional antibiotics and other NCRs in the combined treatment of *E. coli*

The use of multiple antimicrobial agents can simultaneously target different physiological pathways in microbial cells, reducing the likelihood of resistance development and increasing treatment efficacy. To explore this strategy, we assessed the combined antibacterial effects of the NCR169C_17–38_ peptide with several antibiotics - specifically polymyxin B, meropenem, and streptomycin - against *E. coli*. In addition, we tested two short peptide derivatives of NCR335 that showed antimicrobial activities earlier (Farkas et al., 2017). These NCR335N_1–19_ and NCR335C_13–33_ short peptides were previously reported to exhibit antifungal activities against *Candida* and *Cryptococcus* species (Szerencsés et al., 2021; Szerencsés et al., 2025). First, the MBC values of the antibiotics and peptides were determined under our experimental conditions (Table 4), allowing us to define appropriate concentration ranges for subsequent combination studies. Combination treatments were then performed using the standard checkerboard titration method, with each agent co-administered with NCR169C_17–38_. The efficacy of the combinations was evaluated using the Fractional Bactericidal Concentration (FBC) index (FBC = FBC-A + FBC-B) to determine the nature of the interaction between agents (Table 4). Synergistic interactions were observed when NCR169C_17–38_ was combined with polymyxin B and meropenem, whereas its combination with streptomycin resulted in an additive effect. Moreover, the NCR335-derived fragments NCR335N_1–19_ and NCR335C_13–33_, which individually displayed bactericidal activity, also exhibited synergistic effects when combined with NCR169C_17–38_.

**Table 4:** Combined effect of NCR169C_17–38_ with antibiotics and NCR335 peptide fragments against *E. coli*. MBC value of each Drug A is included, NCR169C_17–38_ was consistently used as Drug B.

Drug A	Drug A MBC (µM)	FBC-A (µM)	FBC-B (µM)	FBC	Action
Polymyxin B	0.8	0.125 (0.1)	0.25 (0.4)	0.38	Synergism
Meropenem	200	0.25 (50)	0.25 (0.4)	0.5	Synergism
Streptomycin	3.125	0.5 (1,6)	0.5 (0.8)	1	Additive
NCR335N_1–19_	3.125	0.5 (1.6)	0.01 (0.024)	0.5	Synergism
NCR335C_13–33_	12.5	0.5 (6.25)	0.03 (0.048)	0.5	Synergism

Fractional bactericidal concentration (FBC) index values: < 0.5: synergism; 0.5 < FBC < 1.0: additive; 1.0 < FIC < 2.0: indifferent; >2: antagonism. (µM) corresponds to the concentration of drug A and Drug B for the indicated action.

### 3.4 Biofilm inhibition and eradication by NCR169C_17–38_ in comparison with meropenem and polymyxin B

**Fig. 1:**
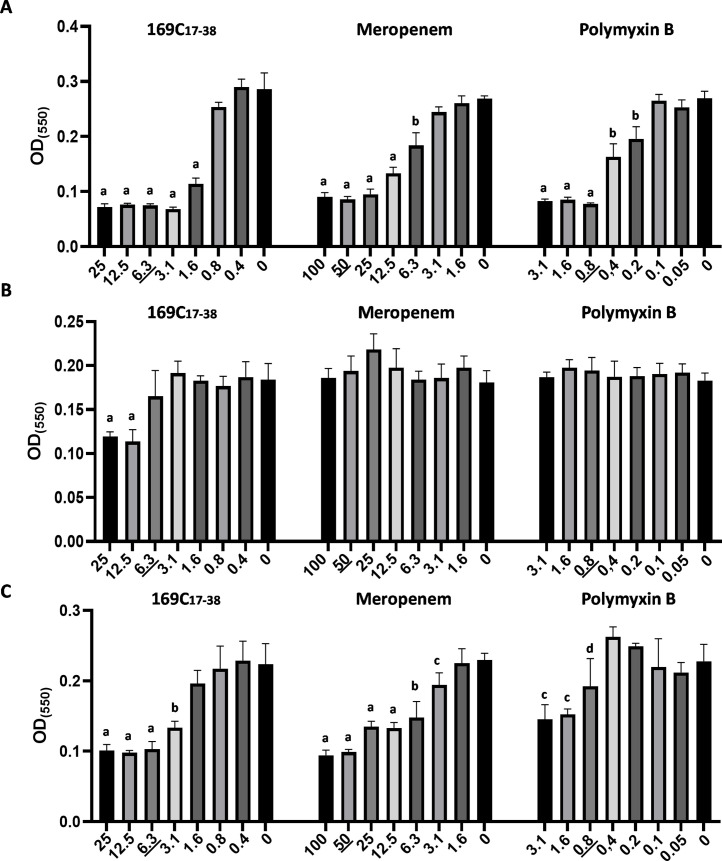
NCR169C_17–38_ effect on biofilm produced by *A. baumannii* compared to antibiotics. After the treatments the biofilm was visualized by Crystal Violet (CV) staining and quantified by measuring OD_550_. The peptide and antibiotics were applied in a series of dilutions, with the MBC values underlined. A. Inhibition of biofilm formation by NCR169C_17–38_, meropenem and polymyxin B. *B* + *C* Biofilm eradication by NCR169C_17–38_, meropenem and polymyxin B after 3 h (B) or 24 h (C) of treatment. The OD (550) values represent the mean ± standard deviation calculated from three independent experiments (a: *p* ≤ 0.0001, b: *p* ≤ 0.001, c: *p* ≤ 0.01, d: *p* ≤ 0.05, unpaired t-test).

We examined the capacity of NCR169C_17–38_ to inhibit biofilm formation by *Acinetobacter baumannii*, a pathogen well known for its robust biofilm production and high level of antibiotic resistance. Initially, we performed the antimicrobial test in MHB with 24 h treatment of NCR169C_17–38_, meropenem and polymyxin B against *A. baumannii* and determined their MBC values as usual (Table 5.). Additionally, at the end of the experiment we determined the quantity of the biofilm and thereby evaluated their ability to inhibit biofilm formation during culturing the bacteria in the presence of different concentrations of the agents (Fig. 1.A). We tested concentrations of NCR169C_17–38_ ranging from 25 μM to 0.4 μM, meropenem from 100 μM to 1.6 μM and polymyxin B from 3.1 μM to 0.05 μM. Biofilm formation by *A. baumannii* was significantly reduced by all three agents when applied at their respective MBC concentrations, with NCR169C_17–38_ and meropenem remaining effective even at lower doses.

**Table 5:** MBC values of NCR169C_17–38_, meropenem and polymyxin B against *A. baumannii* after 24 h of treatment in MHB detected either at the end the biofilm inhibition or at the end of eradication tests against pre-formed biofilm after 3 h or 24 h treatment.

	NCR169C_17–38_	Meropenem	Polymyxin B
Inhibition test after 24 h	6.3 µM	50 µM	0.8 µM
Eradication test after 3 h	6.3 µM	>100 µM	>3.2 µM
Eradication test after 24 h	12.5 µM	>100 µM	>3.2 µM

Bacteria that grow and produce biofilm prior to treatment and thereby located within biofilm exhibit higher resistance against antibiotic treatments. To assess whether the NCR169C_17–38_ peptide or the tested antibiotics could eradicate pre-formed biofilms, *A. baumannii* was first cultured in MHB for 24 h, then treated with NCR169C_17–38_, meropenem or polymyxin B for either 3 h or 24 h, using the same concentration range as in the previous experiment. Both bacterial survival within the pre-formed biofilm and the amount of residual biofilm were assessed. The MBC values obtained in these biofilm eradication assays (Table 5.) indicated that the NCR169C_17–38_ peptide was able to kill bacteria with an efficacy comparable to that observed in the planktonic antimicrobial test. In contrast, neither of the two antibiotics completely eliminated the bacteria within the tested concentration range. Quantification of the residual biofilm at the end of the experiments revealed that, after 3 h of treatment, none of the tested antibiotics were able to eradicate the established biofilms. Only NCR169C_17–38_ was capable of degrading the pre-formed biofilm in this short time, and this effect was observed at concentrations exceeding its MBC (at 12.5 and 25 μM; Fig. 1.B). Interestingly, after 24 h of treatment, all agents demonstrated the ability to degrade biofilms, albeit at different effective concentrations. NCR169C_17–38_ and meropenem were successful even at sub-MBC level (Fig. 1.C), whereas polymyxin B required at least their MBC or higher concentrations to achieve significant biofilm eradication. This 24 h of treatment was repeated on mature biofilm (pre-formed for 72 h) and the results were very similar (Supplementary Fig. 1.). NCR169C_17–38_ peptide was able to kill bacteria with the same efficacy and also could degrade the mature biofilm at sub-MBC level (3.1 μM).

### 3.5 Rapid impact of NCR169C_17–38_ on *E. coli* membrane permeability

**Fig. 2:**
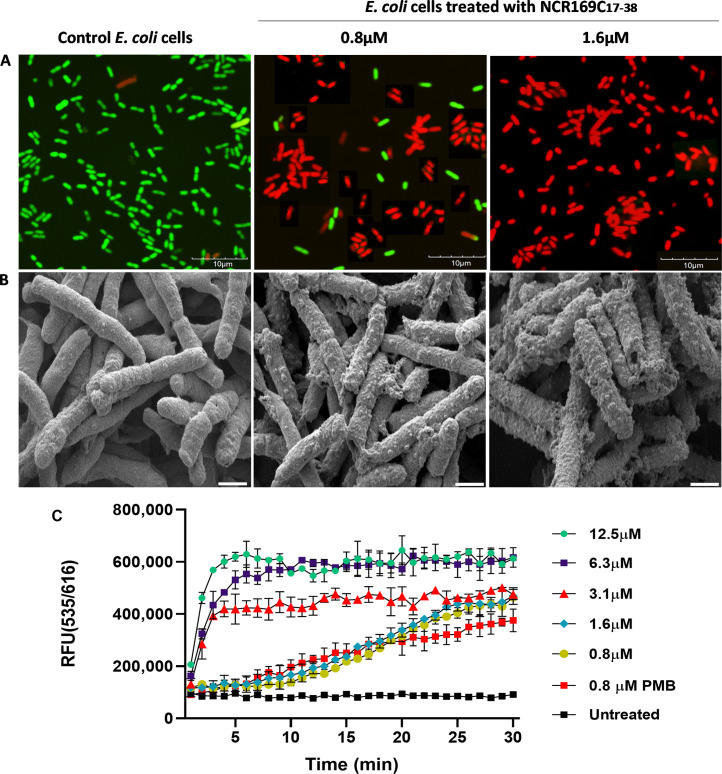
Membrane integrity or damage and morphology of *E. coli* following exposure to various concentrations of the NCR169C_17–38_ peptide. (A) *E. coli* cells were stained with SYTO 9 and PI, and pictures were taken with confocal microscope; scale bar: 10 µm. (B) Cells were treated with the peptide in the absence of fluorescent dyes and imaged using a scanning electron microscope; scale bar: 1 µm. (C) Monitoring of PI uptake in *E. coli* treated with a series of concentrations of NCR169C_17–38_ in Hidex Plate reader. RFU (535/616) = Relative Fluorescence Units (excitation/emission wavelengths). 0.8 µM Polymyxin B (PMB) was used as a control.

Next, we investigated whether the application of NCR169C_17–38_ peptide affects membrane permeability of *E. coli*, and if so, to what extent and how rapidly these changes occur. In the first experiment, *E. coli* cells were exposed to near-MBC concentrations (0.8 and 1.6 µM) of NCR169C_17–38_ for 10 min, followed by staining with SYTO 9 (can enter undamaged living cells staining them green) and propidium iodide (PI, can enter only cells with damaged membrane and staining them red), and examination by confocal microscopy. While untreated control cells exhibited only the green fluorescence of SYTO 9, bacterial cultures exposed to 0.8 µM peptide showed predominantly red-stained cells, with only a few remaining green, indicating rapid membrane disruption as evidenced by PI uptake (Fig. 2.A). At 1.6 µM peptide, all cells appeared red, demonstrating complete and rapid loss of membrane integrity.

To visualize the morphological changes in *E. coli* induced by NCR169C_17–38_, the same treatment was performed without fluorescent dyes. Instead, the cells were fixed and examined using scanning electron microscopy (SEM). Unlike the smooth, intact morphology of untreated cells, *E. coli* cells treated with 0.8 µM NCR169C_17–38_ displayed evident surface roughening and outer membrane blebbing, indicating peptide-induced structural disintegration (Fig. 2.B). At higher peptide concentration (1.6 µM), more pronounced membrane damage was evident, including visible perforations and extrusion of cellular material from ruptured areas.

To get a more precise assessment of how rapidly NCR169C_17–38_ induces membrane damage, we monitored PI uptake in real time. *E. coli* was treated in a microtiter plate with two-fold dilutions of NCR169C_17–38_ (12.5 - 0.8 µM) and PI fluorescence was measured using a plate reader (Fig. 2.C). For comparison, polymyxin B, a well-known membrane-permeabilizing/damaging antibiotic, was included as a control. A rapid rise in fluorescence within 1–2 min was observed upon exposure to NCR169C_17–38_ at MBC or higher concentrations, indicating swift membrane permeabilization. Comparison of PI uptake kinetics of NCR169C_17–38_ and polymyxin B at their respective MBC values showed that NCR169C_17–38_ disrupts bacterial membranes more rapidly and effectively than polymyxin B.

### 3.6 Thermal and serum stability, and lipid-binding specificity of NCR169C_17–38_

Given that NCR169C_17–38_ and NCR169C_17–38_-StrepII displayed comparable antimicrobial activity, we evaluated the stability of NCR169C_17–38_-StrepII at room temperature and heat stress. Peptide dilutions (25, 12.5, 6.3, and 3.1 µM) in PPB were either incubated overnight at room temperature (RT) or heated at 80 °C for 20 min. Dot blot detection of the peptide with an anti-StrepII antibody confirmed its stability (Supplementary Fig. 2.A), and antimicrobial assays against *E. coli* revealed unchanged bactericidal activity relative to freshly prepared controls (both active down to 3.1 μM, Supplementary Fig. 2.B), demonstrating high thermal and storage stability. The serum stability of the peptide was also tested by incubating the NCR169C_17–38_ in human serum for eight hours prior to antimicrobial assay against *E. coli, A. baumannii and S. aureus*. The observed MBC values (3.1, 6.3, and 3.1 μM, respectively) demonstrate that pre-incubation in serum had minimal impact on antibacterial efficacy, suggesting that the peptide remains stable and is not significantly cleaved by human serum proteases.

As shown above, NCR169C_17–38_ causes rapid damage to bacterial membranes. Since membrane structure is largely determined by its lipid composition, we next investigated the ability of NCR169C_17–38_-StrepII to bind membrane lipids *in vitro* using a peptide-lipid overlay assay (Supplementary Fig. 3.A). NCR169C_17–38_-StrepII bound to several anionic lipids, including phosphoinositides, phosphatidic acid, phosphatidylserine, and cardiolipin - a major constituent of bacterial membranes. However, the peptide did not bind to phosphatidylglycerol (PG), a common anionic phospholipid, indicating a degree of specificity in its lipid interactions.

To further confirm the cardiolipin-binding, we performed a liposome binding assay using two phospholipids: cardiolipin (positive in the overlay assay) and DMPC; (dimyristoyl- phosphatidylcholine) that is a derivative of phosphatidylcholine that showed no binding on the lipid strip. Prepared liposomes were incubated with NCR169C_17–38_, then separated by ultracentrifugation. Peptide remaining in the supernatant (sn) fraction indicated no binding, whereas peptide detected in the pellet (p) fraction indicated liposome association. In this setup, NCR169C_17–38_ bound strongly to cardiolipin liposomes but showed no binding to DMPC liposomes (Supplementary Fig. 3.B).

Microscale thermophoresis (MST) was also used to assess the interaction of NCR169C_17–38_ with various lipids. We tested binding of NCR169C_17–38_–5FAM (100 nM) to cardiolipin, DMPC, and a 1:1 DMPC/cholesterol mixture. A binding-induced change in thermophoretic movement, reflected in the normalized fluorescence values with a normalized amplitude change of 15 ‰ and a high signal-to-noise ratio of 9.1 (response evaluated at 10 s) demonstrated a specific interaction (Supplementary Fig. 4.A). To evaluate this interaction, we quantified the binding by titrating with increasing concentrations of cardiolipin (up to 1 µM) (Supplementary Fig. 4.B). The dose–response curve showed a clear thermophoretic shift when lipid was added, indicating formation of a peptide–lipid complex. Nonlinear regression of the binding isotherm yielded a dissociation constant (Kd) of approximately 470 nM, which falls within the expected sub-micromolar range typical for cationic antimicrobial peptides interacting with negatively charged phospholipids (Rainsford et al., 2023). In contrast to cardiolipin, pure DMPC and the 1:1 DMPC/cholesterol mixture showed flat thermophoresis profiles with a normalized amplitude change <5 ‰, indicating the absence of binding under these conditions. These data demonstrate that NCR169C_17–38_ binds cardiolipin with physiologically meaningful affinity, supporting a model in which this peptide preferentially associates with cardiolipin-rich domains of bacterial membranes.

### 3.7 Peptide - nucleic acid interactions and binding dynamics

**Fig. 3:**
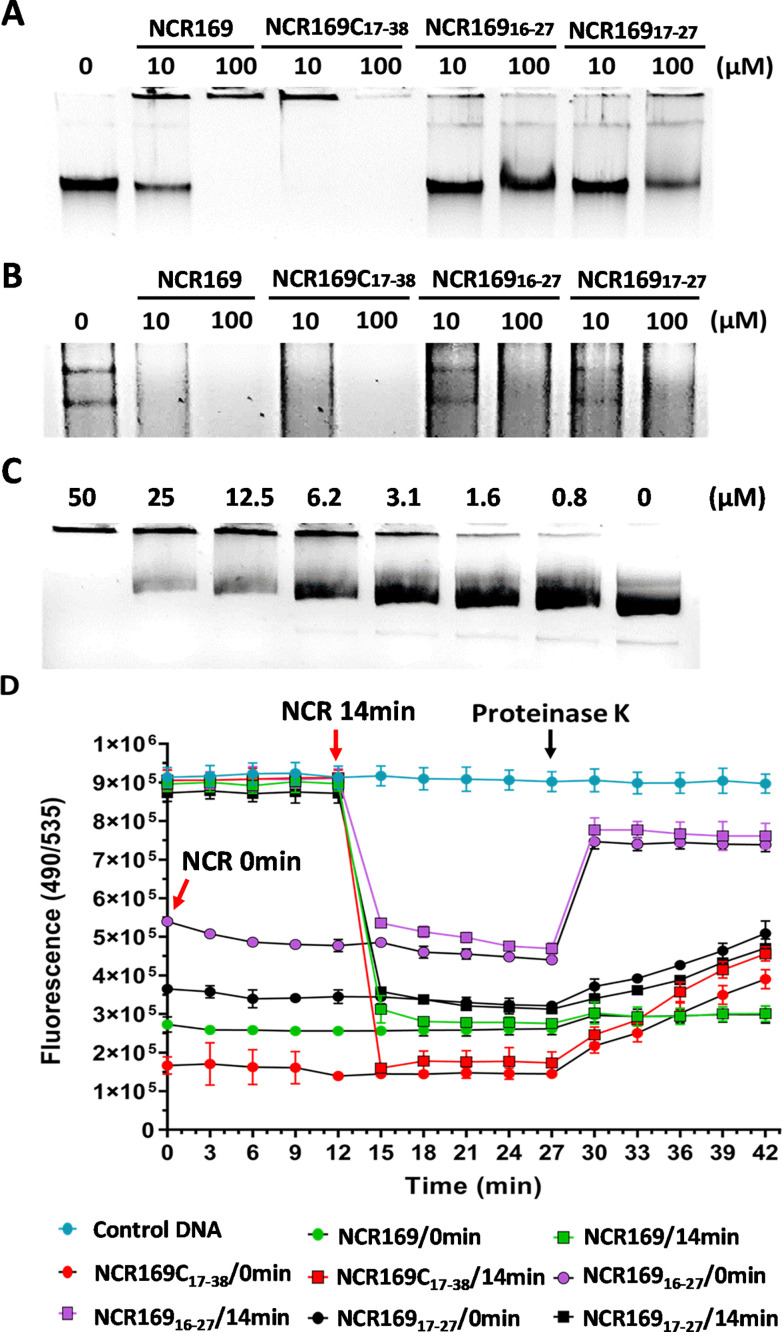
*In vitro* interaction of NCR169C_17–38_, NCR169, NCR169_16–27_ and NCR169_17–27_ with nucleic acids. (A) Gel retardation assay of 100 ng *E. coli* genomic DNA incubated with 0, 10, or 100 µM peptide. (B) Same setup as in (A), but using 100 ng *E. coli* total RNA. (C) Interaction of NCR169C_17–38_ with 250 ng circular plasmid DNA at varying concentrations. (D) Real-time monitoring of DNA binding by fluorescence of SYBR Gold–DNA complexes (excitation 490 nm, emission 535 nm). Peptides (10 µM) were added to 100 ng DNA at 0 or 14 min (red arrows); Proteinase K (1 mg/mL) was added at 27 min (black arrow).

To explore whether the NCR169C_17–38_ peptide has secondary intracellular targets of the bacterial cells beyond the primary membrane disruption, we tested its possible interaction with nucleic acids in gel retardation assays. A pilot experiment indicated strong DNA-binding capacity, prompting us to include the parental NCR169 peptide and the shorter derivatives NCR169_16–27_ and NCR169_17–27_ for comparison (Fig. 3.). When 100 ng *E. coli* genomic DNA was incubated without peptide, it migrated as a clear single band, whereas treatments with 10 or 100 µM peptides resulted in altered patterns (Fig. 3. A). NCR169C_17–38_ showed the strongest effect, completely preventing DNA migration from the well at the lower concentration; at higher concentration, no staining was visible even in the well, suggesting DNA-peptide aggregates made the DNA inaccessible to ethidium bromide. NCR169 also displayed solid binding by fully blocking migration at 100 µM. By contrast, NCR169_16–27_ and NCR169_17–27_ exhibited only weak binding, indicated by faint band shifts and well staining. Similar results were obtained with total *E. coli* RNA (Fig. 3.B). NCR169C_17–38_ and NCR169 completely blocked RNA migration at high concentrations, and at low concentration faint migration signals of RNA could be detected, though the 23S and 16S ribosomal RNA bands disappeared. The shorter derivatives again displayed weaker binding, detectable only at high concentrations. To further probe DNA binding, the interaction of NCR169C_17–38_ with circular plasmid DNA was characterized by using a dilution series of the peptide (Fig. 3.C). Increasing peptide concentrations progressively reduced the quantity of migrating DNA, with complete retention of DNA in the well at higher concentrations, confirming a concentration-dependent interaction.

The DNA-binding kinetics were quantified using SYBR Gold fluorescence (Fig. 3.D). Half of the DNA samples were not treated with peptides at the start, and these displayed the highest fluorescence signals. Samples treated with peptides at time zero showed an immediate decrease in fluorescence, consistent with rapid DNA binding. The extent of signal reduction followed the same hierarchy observed in gel assays: NCR169C_17–38_ > NCR169 > NCR169_16–27_ > NCR169_17–27_. Peptide addition at 14 min similarly caused an abrupt drop in fluorescence to peptide-specific levels. To test if peptide removal would recover the DNA–SYBR Gold fluorescence to control values, Proteinase K was added at 27 min to all reactions. A prompt effect was observed with the shortest NCR169_16–27_ peptide which showed a marked fluorescence increase that stabilized but did not fully reach the original control level. In contrast, fluorescence values in the NCR169 samples remained unchanged, while those of NCR169C_17–38_ or NCR169_17–27_ increased slightly and gradually to the same limited extent. Thus, NCR169_16–27_ proved to be the most sensitive to Proteinase K treatment, NCR169 was resistant, and NCR169C_17–38_ together with NCR169_17–27_ displayed only partial sensitivity. These results demonstrate that NCR169C_17–38_ binds strongly and rapidly to both DNA and RNA in a concentration-dependent manner, forming stable nucleic acid complexes that are only partially reversible by protease treatment.

We validated the ability of NCR169C_17–38_ to interact with bacterial nucleic acids *in vivo* by treating *E. coli* cells with a 5-carboxyfluorescein–labeled peptide (NCR169C_17–38_–5-FAM) for 10 min, followed by staining with 10 µg/mL Hoechst 33,342 to visualize nucleic acids. Confocal microscopy not only revealed clear co-localization of the labeled peptide with cellular DNA (Supplementary Fig. 5), but also confirmed its membrane association, providing direct evidence of the peptide’s dual targeting *in vivo*.

### 3.8 Differential gene expression in *E. coli* exposed to NCR169C_17–38_

Genome-wide transcriptome profiling was performed to examine early global gene expression changes in *E. coli* exposed to a sub-MBC dose of the NCR169C_17–38_ peptide for 20 min in culture medium. Differentially expressed genes (DEGs) were identified by comparing treated and untreated cultures. This analysis revealed significant changes in 503 genes. 450 genes were downregulated, while 53 genes were up-regulated (Supplementary Fig. 6.). 458 DEGs were mapped to 439 annotated *E. coli* genes of the ATCC_8739 genome database, while 45 genes codes for hypothetical proteins.

Kyoto Encyclopedia of Genes and Genomes (KEGG) pathway enrichment analysis was initially performed on the DEGs to identify key pathways that were affected by the NCR169C_17–38_ treatment of *E. coli* (Supplementary Fig. 7.). The highly enriched pathways show an active response from the *E. coli* bacteria in attempt to control the ongoing stress. The affected KEGG pathways can be classified into five major categories as shown in (Supplementary Table 1.): membrane and lipid metabolism, stress response and resistance mechanisms, metabolism and energy production, amino acid and secondary metabolite biosynthesis, and translational and genetic responses. These pathways highlight broad bacterial adaptations, including membrane remodeling (Category 1), altered motility and communication (Category 2), metabolic reprogramming (Category 3), enhanced amino acid and metabolite biosynthesis (Category 4), and increased demands for protein synthesis, transcript processing, and DNA/RNA repair (Category 5). The categorization indicate that peptide treatment triggers coordinated structural, metabolic, and genetic adaptations that promote bacterial survival under stress.

**Fig. 4:**
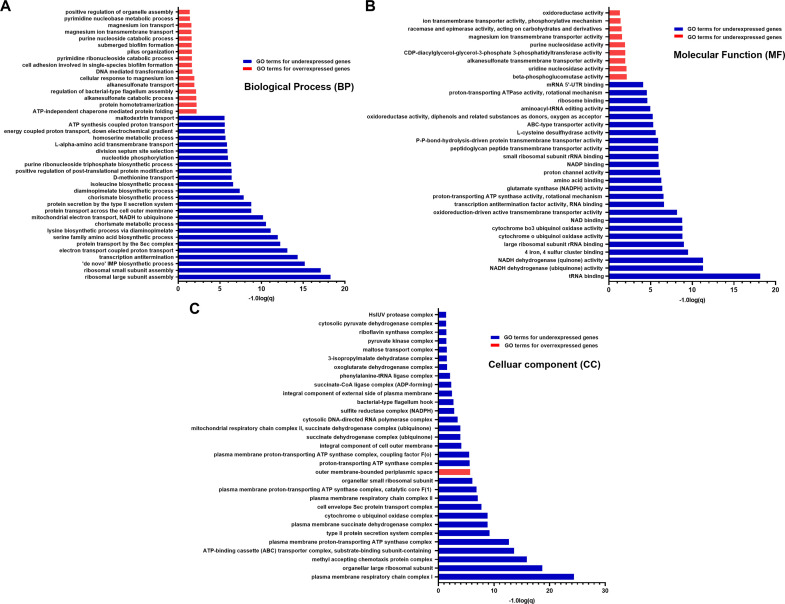
GO enrichment analysis of DEGs in E coli treated with NCR169C_17–38_ annotated in three main categories: (A) biological process, (B) molecular function, and (C) cellular component.

To explore potential biological mechanisms linked to the identified DEGs, GO functional annotation and enrichment analyses were conducted on the upregulated and downregulated genes revealing substantial differences in the biological process (BP), cellular component (CC), and molecular function (MF) terms (Fig. 4A–C). Across all three GO categories, downregulated DEGs were markedly more abundant than upregulated ones. In the BP category, enrichment analysis identified 155 downregulated terms associated with 521 genes, compared to only 16 upregulated terms involving 17 genes. For MF, 155 downregulated terms linked to 415 genes were found, whereas just 9 upregulated terms representing 9 genes were detected. Similarly, in the CC category, 30 downregulated terms encompassing 134 genes were observed, while only a single upregulated term involving 4 genes was enriched. The apparent excess of genes in the GO analysis relative to the transcriptome dataset arises because individual genes are frequently annotated to multiple GO terms. Consequently, the same gene can contribute to several terms, inflating per-term counts even though the overall number of unique genes matches the DEG list.

The peptide appears to induce a broad suppression of bacterial functions, with only a limited subset of genes upregulated, presumably to support cellular rescue and survival. Upregulation of several stress-related genes was observed, including those involved in membrane stress response (*osmB, cpxP, arnC, arnC2, ynbA*), magnesium transport (*mgtA, mgtS*), sulfur metabolism (*ssuA, ssuD*), and oxidative stress (*ycjU, fixX*). *E. coli* also activated genes associated with modulation of host immune responses (*prgK, hcpA*) and biofilm formation (*yehB, yehD, bdM, csgE, ycfS*). Furthermore, upregulation of genes enhancing the translation of stress-related transcripts (*rihB*) or mitigating cellular damage (*yodB, hcpA*) reflects additional adaptive strategies employed by the bacteria to cope with the imposed stress.

The KEGG and GO analyses revealed that NCR169C_17–38_ treatment caused a broad suppression of core bacterial functions. A substantial fraction of downregulated genes was involved in protein synthesis, particularly ribosomal components, translation factors, and aminoacyl-tRNA ligases, indicating a strong inhibition of the translational machinery. Energy production pathways were also markedly affected, with reduced expression of genes associated with ATP synthesis, glycolysis, the tricarboxylic acid cycle, and the electron transport chain. In parallel, key regulators of cell division and transcription were suppressed. Genes involved in cell envelope integrity, including outer membrane transporters, peptidoglycan and fatty acid biosynthesis, and lipid metabolism, showed significantly lower expression, consistent with impaired membrane maintenance. Metabolic pathways supporting amino acid and nucleotide biosynthesis were also downregulated, further reflecting a global shutdown of anabolic processes. Finally, genes governing motility, flagellar assembly, and chemotaxis were strongly repressed, suggesting that adaptive movement and environmental sensing were deprioritized under peptide-induced stress.

Validation of the transcriptome analysis was performed on ten selected DEGs using RT-qPCR, which confirmed the reliability of the sequencing results (Supplementary Fig. 8.).

## 4 Discussion

Our results recognized NCR169C_17–38_ as the most potent AMP derivative of the symbiotic plant peptide NCR169, with consistent bactericidal activity against a wide range of clinically relevant pathogens, including members of the ESKAPE group, as well as *Listeria monocytogenes* and *Salmonella enterica*. Importantly, this activity was maintained under physiological conditions (pH 7.4) and across multiple assay environments.

The original NCR169 peptide, produced during *Medicago truncatula*–*Sinorhizobium meliloti* symbiosis, displayed poor solubility at neutral pH, resulting in variable *in vitro* antimicrobial performance against both Gram-negative and Gram-positive pathogens. The oxidized form (NCR169ox), and the cysteine-free variant NCR169-C_9,15,28,33_/S showed reduced activity at neutral pH. Acidic conditions not only improved peptide solubility but also leveled the efficacy of all of these peptides, highlighting the importance of local physicochemical environments in determining NCR peptide activity. The activity of the cysteine-free variant suggests that, although the presence of cysteines is undoubtedly important for symbiotic function (Horváth et al., 2015), it is not necessary for the antimicrobial effect. Among the various shorter derivatives, the C terminal fragment NCR169C_17–38_ showed the strongest and most consistent bactericidal effect. This 22-amino-acid peptide retains most of the positively charged lysines (six out of seven), both tryptophans, and two short motifs (KIWK and LWYK) resembling the conserved AMP motif XWZX, known to enhance amphiphilicity and membrane interaction (Lee et al., 2018). All shorter peptide fragments resulted in a significant or complete loss of activity in the initially tested PPB buffer. In an earlier study (Isozumi et al., 2021) solving the NMR structure of the NCR169 peptide, the shorter fragment NCR169₁₆_-_₂₇ was previously shown to be active against *E. coli* and *S. meliloti* in HEPES buffer. When comparing the activity of the peptides against the tested bacterial pathogens in different buffers, the MBC values of NCR169_16–27_ were comparable to those of NCR169C_17–38_, only in HEPES buffer. However, even under this condition NCR169_16–27_ peptide exhibited lower activity against L. *monocytogenes* (12.5 µM) and was inactive against *E. faecalis*. In contrast, NCR169C_17–38_ peptide maintained strong antibacterial efficacy across all tested media, including HEPES and PPB buffers, as well as Mueller–Hinton broth, a clinically standardized medium according to the National Committee for Clinical Laboratory Standards (1979), containing divalent cations. This medium might influence the antimicrobial activity of the peptides - consistent with the observation that the antimicrobial activity of NCR plant peptides depends on the test conditions (Farkas et al., 2018). As expected, the presence of these ions slightly increased the MBC values of NCR169C_17–38_ against *E. coli, A. baumannii* and *S. aureaus* (6.3, 6.3, and 12.5 µM, respectively), but this reduction in potency was fully restored by the addition of low concentrations of EDTA, which chelates inhibitory cations. These findings reinforce NCR169C_17–38_ as the minimal unit required for reliable broad-spectrum antimicrobial activity.

Combination assays revealed that NCR169C_17–38_ exhibits synergistic effects with polymyxin B and meropenem, but not with streptomycin, against *E. coli*. Synergism likely arises from complementary mechanisms and suggests that combination strategies could enhance therapeutic efficacy and delay resistance. Similarly, combining NCR169C_17–38_ with NCR335-derived fragments enhanced bactericidal activity, supporting the idea that cocktails of NCR peptides, may represent an effective therapeutic strategy.

One of the most striking findings was the ability of NCR169C_17–38_ to prevent and eradicate *A. baumannii* biofilms. *A. baumannii* is a major multidrug-resistant pathogen whose persistence is largely attributed to its ability to form biofilms on medical devices and host tissues. Within biofilms, bacterial cells are highly protected from antibiotics, immune responses, and environmental stress, often leading to chronic and recurrent infections (Choudhary et al., 2022; Mendes et al., 2023). Besides the NCR169C_17–38_ peptide, both antibiotics - meropenem and polymyxin B - were able to inhibit biofilm formation by *A. baumannii* when added to fresh planktonic cultures. However, when these agents were applied against bacteria within a pre-formed biofilm, only NCR169C_17–38_ was able to significantly eradicate the biofilm after a 3-hour treatment and kill the bacteria in it as effectively as it did in planktonic conditions. After 24 h of treatment, all three antimicrobial agents caused a significant reduction in biofilm when applied at higher concentrations, nonetheless, NCR169C_17–38_ retained significant activity even at sub-MBC concentrations. The MBC values obtained in these biofilm eradication assays indicated that only the NCR169C_17–38_ peptide could kill biofilm-embedded bacteria with an efficacy comparable to that observed in the planktonic cultures. This ability to act against biofilm-associated cells highlights a significant advantage NCR169C_17–38_ over standard treatments.

The potent antimicrobial activity of the NCR169C_17–38_ peptide can be attributed to its rapid and extensive disruption of bacterial membranes. Fluorescence microscopy, scanning electron microscopy, and real-time propidium iodide (PI) uptake assays consistently demonstrated that the bacterial membrane is the primary target of the peptide’s action. Remarkably, the rate of permeabilization induced by NCR169C_17–38_ exceeded that of polymyxin B, a last-resort antibiotic, highlighting the peptide’s potency. Lipid-binding assays revealed preferential binding to cardiolipin over phosphatidylcholine or cholesterol, suggesting a degree of specificity for distinct bacterial membrane components. Since cardiolipin is enriched in bacterial but not mammalian cell membranes, this selectivity likely contributes to both the antimicrobial efficacy and low cytotoxicity of the peptide - a feature of considerable therapeutic value. Consistent with this, NCR169C_17–38_ peptide has been previously shown to be non-cytotoxic to human keratinocyte HaCaT cells (Szerencsés et al., 2021), mouse macrophage J774.2 cells (Szerencsés et al., 2025), and to lack hemolytic activity (Howan et al., 2023).

Across all *in vitro* assays, NCR169C_17–38_ demonstrated antimicrobial potency, membrane permeabilization, and biofilm removal capacities equal to or greater than those of polymyxin B, underscoring its strong therapeutic potential.

Mechanistically, transcriptome profiling revealed that exposure to sublethal concentrations of NCR169C_17–38_ induces profound global transcriptional reprogramming in *E. coli*. >500 genes were differentially expressed within 20 min, with an overwhelming dominance of downregulated genes. This global suppression encompassed key cellular functions, including ribosome biogenesis, translation, energy production, lipid and cell wall biosynthesis, and motility. The inhibition of these essential pathways reflects a rapid and comprehensive shutdown of bacterial metabolism and growth - a hallmark of effective antimicrobial action (Tomasinsig et al., 2004; Jenssen et al., 2006; Kintses et al., 2019). The small subset of upregulated genes was primarily associated with stress response, membrane remodeling, and detoxification pathways, consistent with bacterial attempts to mitigate membrane damage and oxidative stress (Peschel and Sahl, 2006; Andersson et al., 2016; Bechinger and Gorr, 2017). These findings suggest that NCR169C_17–38_ exerts a dual mechanism of action: immediate physical disruption of membrane integrity, followed by secondary intracellular effects that disturb nucleic acid stability and transcriptional control. The capacity of NCR169C_17–38_ to bind nucleic acids further expands its antimicrobial profile beyond mere membrane disruption. DNA and RNA binding assays demonstrated strong, concentration-dependent interactions, forming stable peptide–nucleic acid complexes that only partially dissociate upon protease treatment. Such interactions likely contribute to the observed transcriptional silencing and metabolic arrest, providing an additional layer of antibacterial activity. Together with its preferential binding to cardiolipin—a major anionic lipid of bacterial membranes but absent from mammalian membranes—this selectivity underpins both its potency and safety.

From a pharmacological perspective, NCR169C_17–38_ combines several highly desirable attributes: broad-spectrum antimicrobial and antifungal (Szerencsés et al., 2021, 2025) activity, rapid bactericidal kinetics, antibiofilm capacity, synergism with antibiotics, high physicochemical stability, and stability in human blood serum alongside the lack of cytotoxicity to mammalian cells. These properties position it as a strong candidate for therapeutic development, either as a standalone antimicrobial or as an adjuvant to enhance antibiotic efficacy. Its stability at elevated temperatures and in storage further supports practical applicability in clinical or agricultural contexts.

## 5 Conclusions

Our findings classify NCR169C_17–38_ peptide as a stable, broad-spectrum AMP with rapid bactericidal action, synergistic potential with antibiotics, and unique antibiofilm activity. Its dual targeting of membranes and nucleic acids contributes to the collapse of essential cellular processes, explaining its robust antimicrobial efficacy. Together with its previously demonstrated antifungal activity and lack of cytotoxicity effect toward different mammalian cell lines, NCR169C_17–38_ represents a promising candidate for development as a next-generation antimicrobial agent. Future work should evaluate its *in vivo* activity and potential as part of combination therapies, particularly for biofilm-associated and multidrug-resistant infections.

## Funding

This work was funded by the Hungarian National Office for Research, Development and Innovation (NKFIH) through the Frontline Research grant KKP129924 to ÉK, the STARTING_151207 grant to RML, the 2021–1.2.6-TÉT-IPARI-MA grant to GE, and the Balzan research grant to ÉK.

## Ethics approval

Not applicable.

## Declaration of generative AI and AI-assisted technologies in the writing process

During the preparation of this work, no generative AI was used to produce original content. However, the authors employed ChatGPT-4.0 solely to improve the grammar, clarity, and readability of the manuscript, as English is not their native language. All text generated with AI assistance was thoroughly reviewed, revised, and verified by the authors to ensure accuracy, coherence, and consistency with the intended meaning.

## CRediT authorship contribution statement

**Mohamad Anas Al Bouni:** Investigation, Validation, Visualization, Data curation, Formal analysis, Writing – review & editing. **Rui M. Lima:** Investigation, Data curation, Visualization, Writing – original draft, Funding acquisition. **Sándor Jenei:** Investigation. **Hilda Tiricz:** Investigation. **Edit Tímár:** Investigation, Writing – original draft. **Ildikó Domonkos:** Investigation. **Éva Kondorosi:** Conceptualization, Supervision, Project administration, Writing – review & editing, Funding acquisition. **Gabriella Endre:** Conceptualization, Supervision, Project administration, Methodology, Writing – review & editing, Funding acquisition.

## Declaration of competing interest

Authors declare that there are no known competing financial interests or personal relationships that could have appeared to influence the work reported.

## Data Availability

The transcriptome data generated in this study have been deposited in the NCBI Gene Expression Omnibus (GEO) under accession number GSE312438.

## Appendix Supplementary materials

Image, application 1
