# Supplementary material for: A plant-derived antimicrobial peptide with multiple mechanisms of action exhibiting antibacterial and antibiofilm activities comparable to or superior to polymyxin B
Source: Curr Res Microb Sci. 2025 Dec 17;10:100535. doi: 10.1016/j.crmicr.2025.100535 (PMC12811501; doi:10.1016/j.crmicr.2025.100535)
Supplement: Supplementary file 1 [file mmc1.docx]

****Supplementary Material for****

****A plant-derived antimicrobial peptide with multiple mechanisms of action exhibiting antibacterial and antibiofilm activities comparable to or superior to polymyxin B****

Mohamad Anas Al-Bouni^1,2,#^, Rui M. Lima^1,#^, Sándor Jenei^1^, Hilda Tiricz^1^, Edit Tímár^1^, Ildikó Domonkos^1^, Éva Kondorosi^1^, and Gabriella Endre^1,^*

^1^Institute of Plant Biology, HUN-REN Biological Research Centre, Szeged, Hungary

^2^Doctoral School of Biology, Faculty of Science and Informatics, University of Szeged, Szeged, Hungary

***** Correspondence: endre.gabriella@brc.hu

**#** These authors contributed equally to this work.


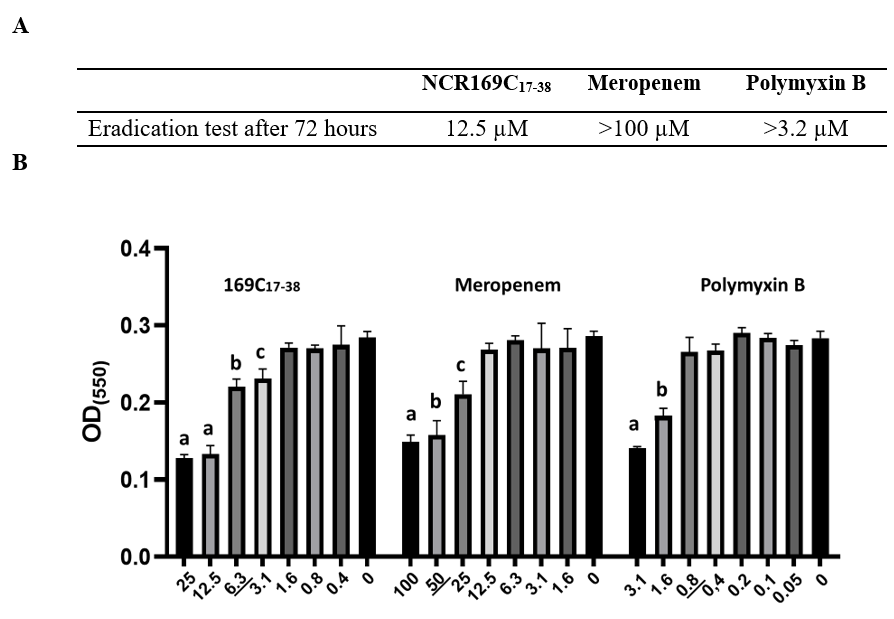


**Supplementary Figure 1.** NCR169C_17-38_ effect on pre-formed mature biofilm produced by *A. baumannii* for 72 hours compared to antibiotics.

(A) MBC values of NCR169C_17-38_, meropenem and polymyxin B against *A. baumanni* after 24 h of treatment, detected at the end of eradication tests against mature biofilm pre-formed for 72 h. (B) After the treatments the biofilm was visualized by Crystal Violet (CV) staining and quantified by measuring OD_550_. The peptide and antibiotics were applied in a series of dilutions, with the MBC values underlined. Biofilm eradication by NCR169C_17-38_, meropenem and polymyxin B after 24 hours of treatment. The OD (550) values represent the mean ± standard deviation calculated from three independent experiments (a: p ≤ 0.0001, b: p ≤ 0.001, c: p ≤ 0.01, d: p ≤ 0.05, unpaired t test).


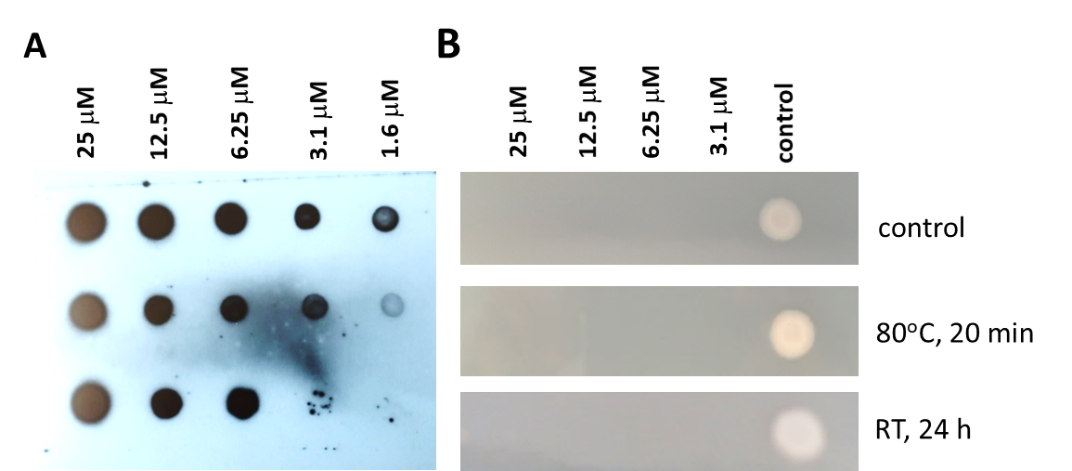


Supplementary Figure 2. NCR169C_17-38_-StrepII shows high heat stability.

(A) Detection of NCR169C_17-38_-StrepII with anti-StrepII antibodies on a dot blot.

(B) NCR169C_17-38_-StrepII remains active against *E. coli* even at 3.1 μM after 20 minutes of treatment at 80 °C or incubation at room temperature for 24 hrs. Bacterial viability was assessed by plating 5 μL aliquots after treatment on LB agar. Only non-treated control cells could grow.


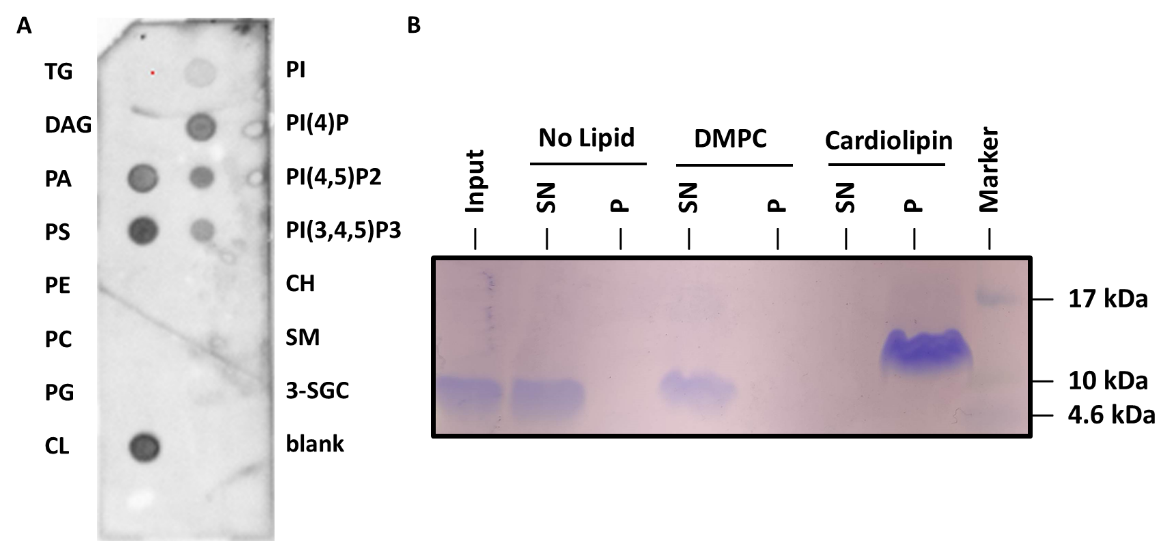


Supplementary Figure 3. Detecting lipid-binding ability of NCR169C_17-38_. (A) NCR169C_17-38_-StrepII was overlaid onto commercially available lipid strip membrane. The peptide bound to lipids was detected with anti-StrepII-antibody. TG, Triglyceride; DAG, Diacylglycerol; PA, Phosphatidic acid; PS, Phosphatidylserine; PE, Phosphatidylethanolamine; PC, Phosphatidylcholine; PG, Phosphatidylglycerol; CL, Cardiolipin; PI, Phosphatidylinositol; PI4P, Phosphatidylinositol-4-phosphate; PI(4,5)P2, Phosphatidylinositol-4,5-bisphosphate; PI(3,4,5)P3, Phosphatidylinositol-3,4,5-trisphosphate; CH, Cholesterol; SM, Sphingomyelin; 3-SGC, 3-sulfogalactosylceramide. (B) liposome binding assay. 20 μM of NCR169C_17-38_ peptide was incubated with buffer only (No lipid), 2 mM dimyristoyl- phosphatidylcholine (DMPC) liposomes, or 2 mM Cardiolipin liposomes. After incubation and ultra-centrifugation, the supernatant and pellet fractions were analyzed by Glycine-SDS-PAGE. Input is also no lipid sample but before ultra-centrifugation.


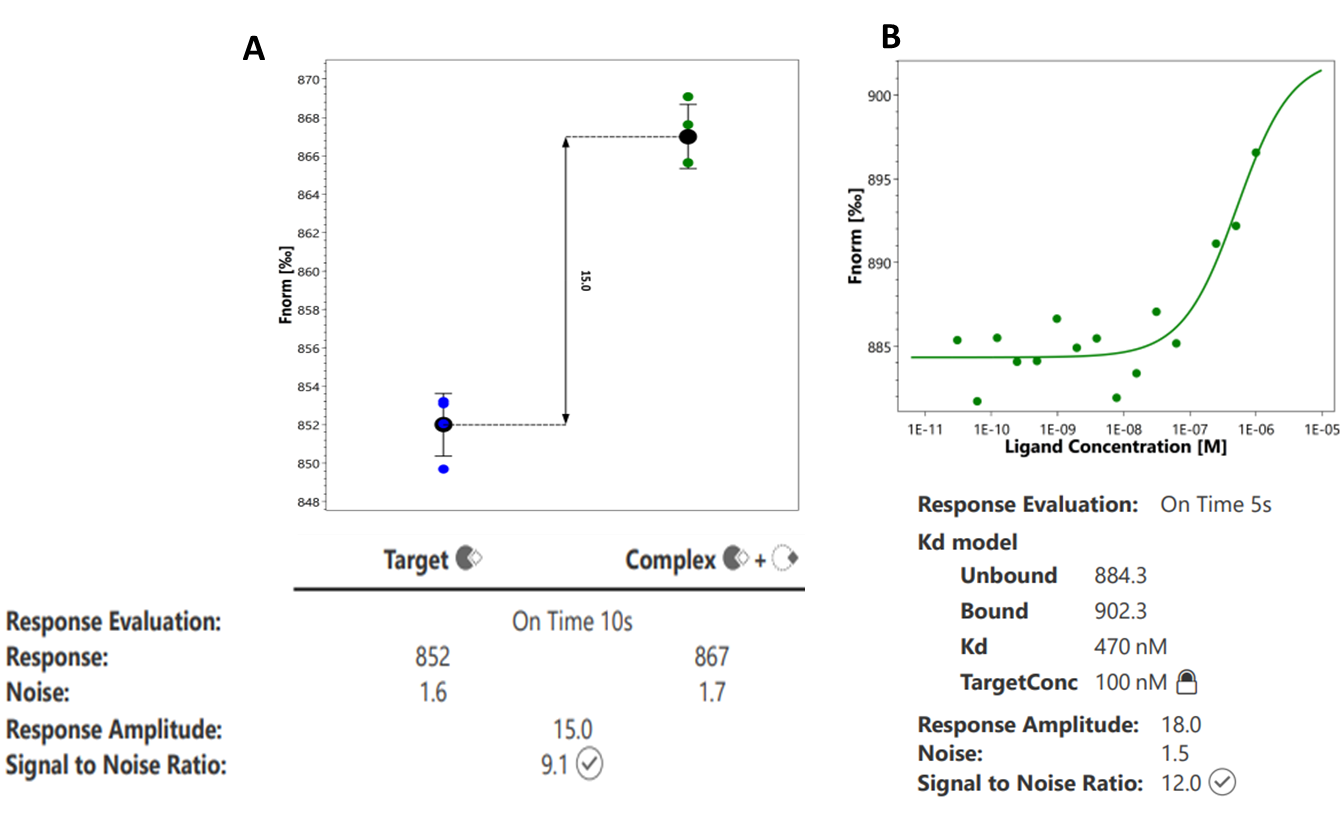

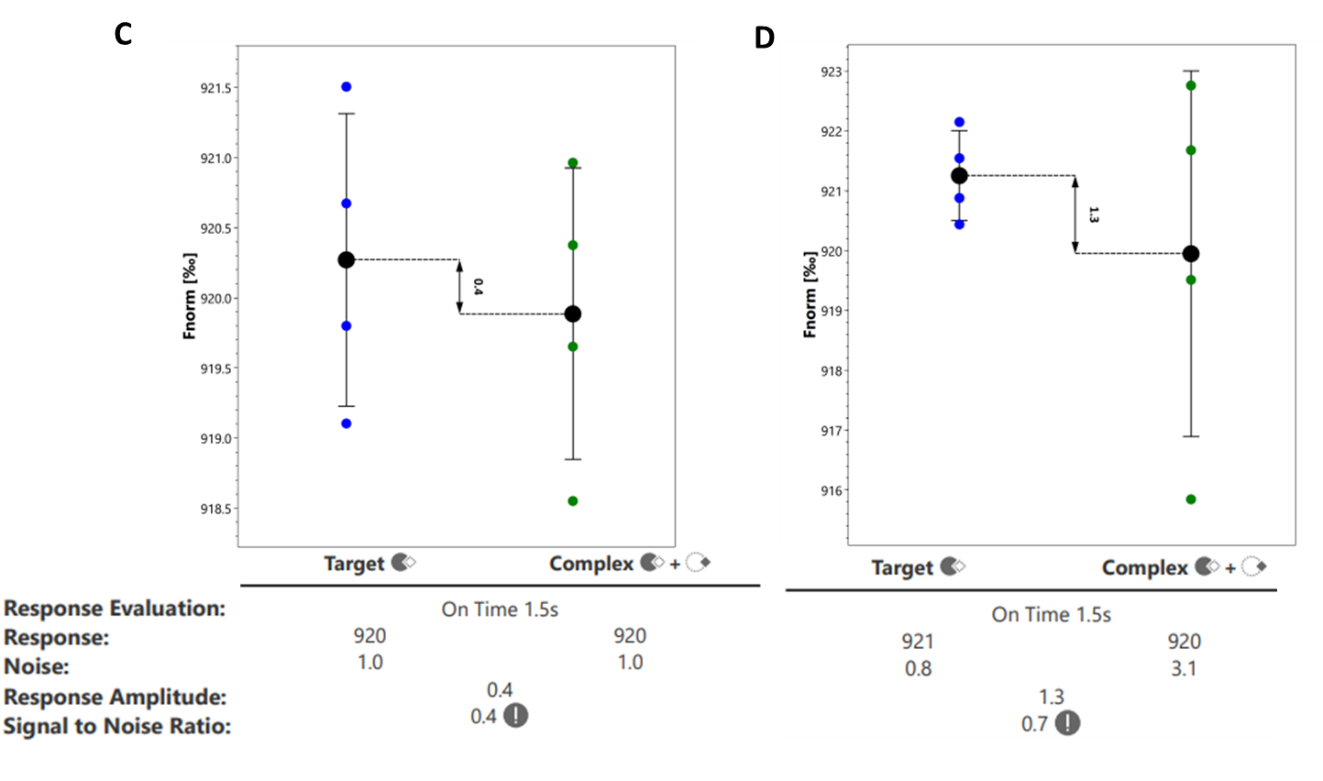


**Supplementary Figure** **4**. Quantification of the binding affinities of NCR169C_17-38_ by Microscale thermophoresis assay. Binding-induced change in molecular movement under a temperature gradient was shown by the calculated normalized fluorescence values, expressed as a thousandth (Fnorm) between 100 nM NCR169C_17-38_ and 1 µM of Cardiolipin (A), DMPC (C) and 1:1 DMPC/Cholesterol (D).The affinity of the Cardiolipin binding to the peptide was measured (B) using a gradient of 2-fold dilution starting from 1 µM Cardiolipin.


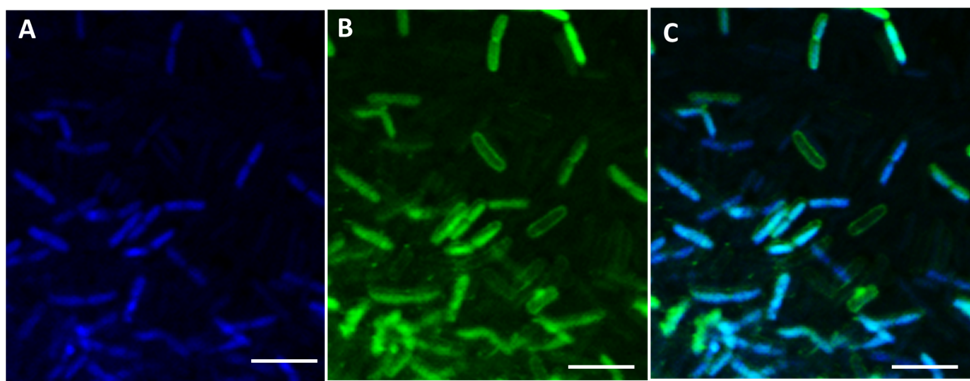


**Supplementary Figure 5**. Localization of NCR169C_17-38_-5-FAM peptide in *E. coli*. Cells were exposed to 1.6 µM NCR169C_17-38_-5-FAM peptide (green) and counterstained with 10 µg/mL Hoechst 33342 (blue) to visualize DNA. The image shows that the peptide co-localize with bacterial nucleic acids and also bind to membrane. Scale bars: 5µm


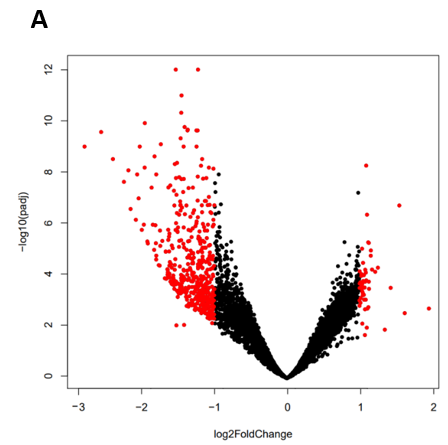


Supplementary Figure 6. Volcano plot showing differentially expressed genes in *E coli* treated with NCR169C_17-38_.


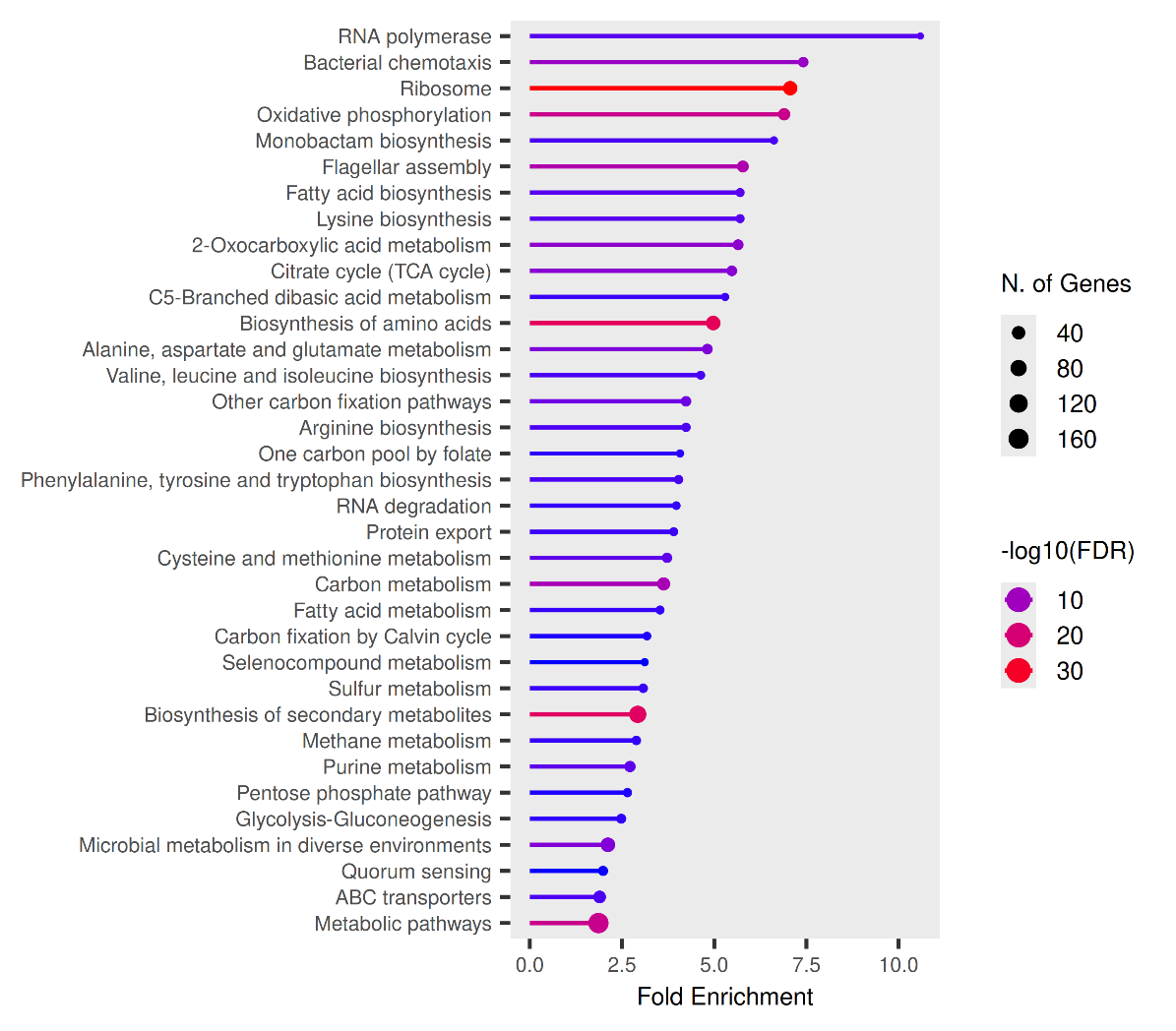


Supplementary Figure 7. KEGG pathway terms associated with the (DEGs) in *E coli* treated with NCR169C_17-38_ on the y-axis and Fold enrichment on the x-axis: Circle size indicates the number of genes involved in the pathway, the bigger the circle, the more the genes. The color indicates significance; light color shows high significance, while dark color shows low significance.


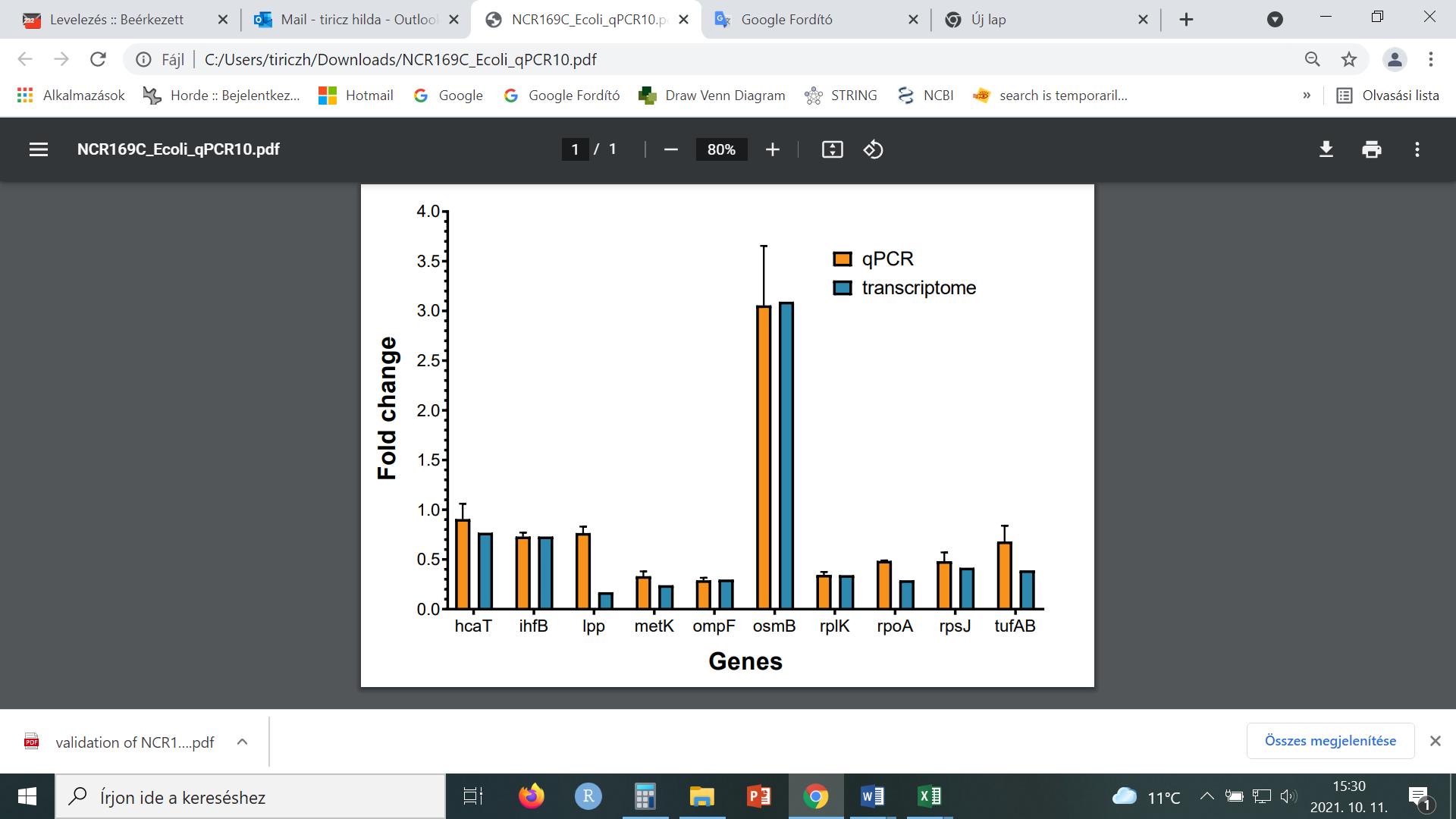


**Supplementary Figure 8**. Validation of transcriptomic results by RT-qPCR reactions. The RT-qPCR values are the result of three technical replicates and the diagram indicates the standard deviation of the values.

**Supplementary Table 1.** The (KEGG) pathway terms identified in our data grouped into five main categories, and the number of genes enriched in each pathway.

| **Category** | **KEGG Pathway ID** | **No. of Genes** |
| --- | --- | --- |
| **1. Membrane and Lipid Metabolism** | **Fatty acid biosynthesis (eco00061) and** **C5-Branched dibasic acid metabolism (eco00660)** – Compensatory membrane remodeling. | 7-5 |
|  | **Fatty acid metabolism (eco01212)** – Adaptations in lipid processing. | 7 |
|  | **ABC transporters (eco02010)** – Changes in nutrient and ion transport. | 32 |
|  | **Bacterial secretion system (eco03070)** – Impact on protein transport. | 7 |
| **2. Stress Response and Resistance Mechanisms** | **Oxidative phosphorylation (eco00190)** – Suggests oxidative stress. | 28 |
|  | **Sulfur metabolism (eco00920)** – Potential response to reactive oxygen species. | 9 |
|  | **Quorum sensing (eco02024)** – Bacterial communication for survival. | 12 |
|  | **Bacterial chemotaxis (eco02030)** – Limiting movement in response to stress. | 14 |
|  | **Flagellar assembly (eco02040)** – Motility changes due to membrane stress. | 24 |
| **3. Metabolism and Energy Production** | **Carbon metabolism (eco01200)** – Central energy metabolism shift. | 37 |
|  | **Citrate cycle (TCA cycle) (eco00020)** – Changes in energy production. | 15 |
|  | **Glycolysis/Gluconeogenesis (eco00010)** – Energy source adjustments. | 11 |
|  | **Pentose phosphate pathway (eco00030)** – NADPH production for stress defense. | 8 |
|  | **Butanoate metabolism (eco00650)** – Energy balance shifts. | 7 |
|  | **Metabolic pathways (eco01100) and Microbial metabolism in diverse environments (eco01120)** – Necessary metabolic changes during the stress. | 163-54 |
| **4. Amino Acid and Secondary Metabolite Biosynthesis** | **Biosynthesis of amino acids (eco01230)** – Central pathway integrating multiple amino acid biosynthesis particularly under stress conditions. | 54 |
|  | **Biosynthesis of secondary metabolites (eco01110)** – Potential antibiotic resistance mechanisms. | 97 |
|  | **Aminoacyl-tRNA biosynthesis (eco00970)** – Adjustments in translation. | 17 |
|  | **Valine, leucine, and isoleucine biosynthesis (eco00290)** – Branched-chain amino acid biosynthesis critical for stress adaptation. | 7 |
|  | **Cysteine and methionine metabolism (eco00270) and Glycine, serine, and threonine metabolism (eco00260)** – Protein synthesis shifts vital for stress adaptation. | 13-8 |
|  | **Lysine biosynthesis (eco00300)**, **Arginine biosynthesis (eco00220)** – Essential amino acids for survival. | 7-8 |
|  | **2-Oxocarboxylic acid metabolism (eco01210)** – Central metabolic hub linking amino acid biosynthesis and energy production. | 16 |
|  | **Phenylalanine, tyrosine, and tryptophan biosynthesis (eco00400)** – Aromatic amino acids signaling molecules important in stress adaptation | 8 |
| **5. Translational and Genetic Responses** | **Ribosome (eco03010)** – Increased protein synthesis demand. | 52 |
|  | **RNA degradation (eco03018)** – Processing of stress-induced transcripts. | 6 |
|  | **Protein export (eco03060)** – Adaptations in stress protein secretion. | 7 |
|  | **Monobactam biosynthesis (eco00261)**, **Novobiocin biosynthesis (eco00401)** – Antibiotic resistance responses. | 5-2 |
|  | **Biosynthesis of cofactors (eco01240)** – Enzyme cofactor adjustments. | 21 |
|  | **One carbon pool by folate (eco00670)** – Nucleotide biosynthesis adaptation. | 5 |
|  | **Purine metabolism (eco00230)** – Nucleotide repair responses. | 20 |
|  | **Methane metabolism (eco00680)** – Potential alternative metabolic shifts. | 9 |
|  | **Selenocompound metabolism (eco00450)** – Possible stress response. | 5 |

**Supplementary Table 2**. qPCR primers for validation of the mRNA sequencing data.

| **Gene** | **Primer name** | **Primer sequence** |
| --- | --- | --- |
| *lpp* | lppcoli_qPCR_F | TGGTACTGGGCGCGGTAATC |
|  | lppcoli_qPCR_R | AGCAGCCTGAACGTCGGAAC |
| *metK* | metKcoli_qPCR_F | GCCAGCACCTATCACCTATG |
|  | metKcoli_qPCR_R | AGCGATTTCTGGTCGATCTC |
| *hcaT* | hcaTcoli_qPCR_F | GCTGCTCGGCTTTCTCATCC |
|  | hcaTcoli_qPCR_R | CCAACCACGCTGACCAACC |
| *cysG* | cysGcoli_qPCR_F | TTGTCGGCGGTGGTGATGTC |
|  | cysGcoli_qPCR_R | ATGCGGTGAACTGTGGAATAAACG |
| *ihfB* | ihfBcoli_qPCR_F | GCGGTTTCGGCAGTTTCT |
|  | ihfBcoli_qPCR_R | CGCAGTTCTTTACCAGGTTT |
| *tufA/B* | tufA/Bcoli_qPCR_F | TCGTTCGTGGTTCTGCTCTG |
|  | tufA/Bcoli_qPCR_R | TCAATCGCACGCTCTGGTTC |
| *ompF* | ompFcoli_qPCR_F | TGGCGGCGTTGCTACCTATC |
|  | ompFcoli_qPCR_R | TTAGAGCGGCGTGCAGTGTC |
| *rplK* | rplKcoli_qPCR_F | ACCGTTTACGCTGACCGTTC |
|  | rplKcoli_qPCR_R | TTGGTCTGCGCGATTTCCTG |
| *osmB* | osmBcoli_qPCR_F | AAATGACCGCGGCTGTTCTG |
|  | osmBcoli_qPCR_R | GCTGCACCACCTAATGTACC |
| *rpsJ* | rpsJcoli_qPCR_F | ATCAAGCAACCGCGGAAATC |
|  | rpsJcoli_qPCR_R | ATGTCAACCAGACGCAAGTG |
| *rpoA* | rpoAcoli_qPCR_F | GCCACCTGACCGATGAGAAC |
|  | rpoAcoli_qPCR_R | TACGCTCCACAGGGCTGTAG |
